# Comparative patterns of modified nucleotides in individual tRNA species from a mesophilic and two thermophilic archaea

**DOI:** 10.1261/rna.077537.120

**Published:** 2020-12

**Authors:** Philippe Wolff, Claire Villette, Julie Zumsteg, Dimitri Heintz, Laura Antoine, Béatrice Chane-Woon-Ming, Louis Droogmans, Henri Grosjean, Eric Westhof

**Affiliations:** 1 Architecture et Réactivité de l'ARN, Institut de Biologie Moléculaire et Cellulaire du CNRS, Université de Strasbourg, F-67084, Strasbourg, France; 2 Institut de Biologie Moléculaire des Plantes du CNRS, Université de Strasbourg, F-67084, Strasbourg, France; 3 Laboratoire de Chimie Biologique, Université Libre de Bruxelles, Institut Labiris, B-1070, Belgium

**Keywords:** mass spectrometry, Archaea, tRNA, modifications, hyperthermophiles

## Abstract

To improve and complete our knowledge of archaeal tRNA modification patterns, we have identified and compared the modification pattern (type and location) in tRNAs of three very different archaeal species, *Methanococcus maripaludis* (a mesophilic methanogen), *Pyrococcus furiosus* (a hyperthermophile thermococcale), and *Sulfolobus acidocaldarius* (an acidophilic thermophilic sulfolobale). Most abundant isoacceptor tRNAs (79 in total) for each of the 20 amino acids were isolated by two-dimensional gel electrophoresis followed by in-gel RNase digestions. The resulting oligonucleotide fragments were separated by nanoLC and their nucleotide content analyzed by mass spectrometry (MS/MS). Analysis of total modified nucleosides obtained from complete digestion of bulk tRNAs was also performed. Distinct base- and/or ribose-methylations, cytidine acetylations, and thiolated pyrimidines were identified, some at new positions in tRNAs. Novel, some tentatively identified, modifications were also found. The least diversified modification landscape is observed in the mesophilic *Methanococcus maripaludis* and the most complex one in *Sulfolobus acidocaldarius*. Notable observations are the frequent occurrence of ac^4^C nucleotides in thermophilic archaeal tRNAs, the presence of m^7^G at positions 1 and 10 in *Pyrococcus furiosus* tRNAs, and the use of wyosine derivatives at position 37 of tRNAs, especially those decoding U1- and C1-starting codons. These results complete those already obtained by others with sets of archaeal tRNAs from *Methanocaldococcus jannaschii* and *Haloferax volcanii*.

## INTRODUCTION

Transfer RNAs are the most modified RNA molecules in terms of number of modified positions and diversity of chemical modifications. A whole gamut of modification enzymes had to differentially evolve in the three domains of life to mature properly tRNAs (Helm and Alfonso 2014; Boccaletto et al. 2018). In short, tRNA modifications play two central roles. The first one is to guarantee the maintenance of the uniqueness and stability of the tRNA architectural fold (Helm 2006; Motorin and Helm 2010, 2011), a requirement for proper recognition by key factors like aminoacyl-tRNA synthetases (Giégé and Springer 2016), the Elongator complex (Karlsborn et al. 2014), and ribosomal subunits (Selmer et al. 2006). The modifications occur in the whole body of the tRNA but especially in the elbow created by the intricate contacts formed between the D- and T-loops (Machnicka et al. 2014). The second main role played by tRNA modifications is to guarantee fidelity and efficiency during ribosomal translation at the decoding site, thereby participating in the regulation of the translational activity and the control of proteostasis (Pollo-Oliveira and de Crécy-Lagard 2019). These modifications occur mainly in the extended anticodon loop of tRNAs (Yarus 1982). Their roles are (i) to maintain a conformation of the anticodon loop preorganized for pairing with the mRNA codon in the A site (Vendeix et al. 2012); (ii) to stabilize the weak AU-rich codon/anticodon pairs (Grosjean and Westhof 2016); (iii) to avoid miscoding (e.g., Met/Ile or Trp/Stop) (Cantara et al. 2013); (iv) to allow the decoding of purine-ending codons in split codon boxes by promoting unusual base-pairings that fit within the decoding ribosomal grip (Rozov et al. 2016). Although the nature and number of the modifications vary considerably between the three domains of life, the great majority of the tRNA positions that are modified are highly conserved throughout phylogeny.

Unfortunately, the complete patterns of tRNA modifications are known for only a limited number of species (e.g., *E. coli* in Bacteria or *S. cerevisiae* in Eukarya). In Archaea, although studied for a long time, the landscape of tRNA modifications remained disperse, and known only for a few particular tRNA isoacceptors or bulk cellular tRNAs (see for examples: Kuchino et al. 1982; Edmonds et al. 1991; Tomikawa et al. 2013). Only in the cases of *Haloferax volcanii* (Gupta 1984, 1986; Grosjean et al. 2008a) and very recently in the case of *Methanocaldococcus jannaschii* (Yu et al. 2019), the modification landscape of a complete set of cellular tRNAs has been elucidated. These data reveal that a few modifications are unique to certain archaea, while others are present in most, if not all archaeal species studied so far. Among the archetypal ones are a N1-methylated pseudouridine at position 54 instead of a thymine (Pang et al. 1982; Gupta 1984), the presence of archaeosine, or 7-formamidino-7-deazaguanosine, at position 15 (Watanabe et al. 1997), certain wyosine derivatives like imG, imG2 and mimG at position 37 (de Crécy-Lagard et al. 2010) and the presence at position 34 of tRNA-Ile of agmatidine, a modified C where the carbonyl group is replaced by decarboxy-arginine (Ikeuchi et al. 2010; Mandal et al. 2010).

Here, we examined the landscapes of tRNA modifications in three archaeal species with very different evolutionary history. Two belong to the Kingdom *Euryarchaeota*: *Methanococcus maripaludis*, a methane-producing anaerobic mesophilic archaeon belonging to the same Methanococcales clade as *M. jannaschii* (but the latter is thermophilic) and *Pyrococcus furiosus*, an anaerobic hyperthermophilic archaeon belonging to the Thermococcales clade (Forterre 2015). The third species studied here is *Sulfolobus acidocaldarius* (an obligate aerobic, acidophilic, sulfur-oxidizing thermophile that belongs to the Sulfolobales clade of the *Crenarchaeota* Kingdom). The last two archaea therefore belong to families not yet systematically analyzed and *S. acidocaldarius* is the first crenoarchaeon for which the modification landscape is reported. Further, all euryarchaeal tRNAs analyzed so far are from anaerobic microorganisms (with thermophilic or hyperthermophilic character), while *S. acidocaldarius* is an aerobic moderate thermophile.

As performed earlier in the case of *H. volcanii* (Gupta 1984, 1986), two-dimensional gel electrophoresis was used to separate individual tRNA isoacceptors from purified bulk cellular tRNAs of each of the three archaea, allowing the analysis of 79 cellular tRNAs out of a total of 116 theoretical corresponding tDNA genes (Chan and Lowe 2009, 2016). In-gel digestion by specific nucleases followed. The resulting tRNA digests, for which sequences were deduced from their known genomes, were then separated by chromatography and analyzed by mass spectrometry. The analysis of modified nucleosides of bulk tRNAs was also performed and the results compared with those obtained from the analysis of oligonucleotide sequences. Altogether, our results support the idea that, despite the fact that Archaea share typical modified nucleotides present in both Bacteria and/or Eukarya, they also display unique and specific modifications.

## RESULTS

### tRNA genes and their cellular mature products

In the GtRNAdb (Chan and Lowe 2009, 2016), there are 37 genes coding for tRNAs in *M. maripaludis* and 46 in both *P. furiosus* and *S. acidocaldarius*, all predicted with excellent scores using tRNAscan-SE 2.0 (Lowe and Eddy 1997). In *S. acidocaldarius*, 50 tRNA genes are predicted but four have scores below 45.0 and would be considered as pseudogenes (Chan and Lowe 2009, 2016). Based on genomic information, in *M. maripaludis*, there are only two tRNA genes corresponding to 4-codon boxes and only one gene corresponding to 2-codon boxes, except for Asp, Glu, and Lys where there are two isoacceptors. In *P. furiosus* and *S. acidocaldarius,* there are three genes coding for isoacceptors in 4-codon boxes and only one in 2-codon boxes, except again for Glu, Lys, but also Gln, Ile, Arg(AGR), and Leu(UUR) (all codons of 2/3-codon boxes ending with R3 and thus decoded by Y34-containing tRNAs). In the three species, there is one gene coding for tRNA-Met^i^ and another one for tRNA-Met, except in *M. maripaludis*, where there are two copies of tRNA-Met^i^. As in all other archaeal genomes sequenced so far, there is no gene coding for A34-containing tRNA. Remarkably, in *M. maripaludis*, the C34-containing tRNAs are absent in both 4- and 2-codon boxes except of course in tRNA-Ile(CAU), tRNA-Met and tRNA-Met^i^(CAU), and tRNA-Trp(CCA). Thus, besides these five particular tRNAs, all the other tRNA anticodon triplets start with either G34 or U34. In contrast, in the two thermophilic archaea, *P. furiosus* and *S. acidocaldarius,* the C34-containing tRNAs are present, which explains the increase from 37 to 46 naturally occurring tRNA genes. These distributions follow the sparing strategies in Archaea described by Grosjean et al. (2010). The percentage of GC-content increases from *M. maripaludis* (34%) to *S. acidocaldarius* (37.5%) and to *P. furiosus* (41.1%). The codon usage is such that U3- and A3-ending codons are highly preferred (Nakamura et al. 2000; Emery and Sharp 2011; Nayak 2013). This is particularly striking in the mesophilic *M. maripaludis* where U3- and A3-ending codons are decoded solely by G34-tRNAs and U34-tRNAs, respectively. In *M. maripaludis*, the percentage of GC-content at the third position is 25.6%, while it is 28.8% in *P. furiosus* and as high as 39.3% in *S. acidocaldarius*. Such differences in decoding strategies have consequences on the modification identity found at position 34 and in the extended anticodon stem–loop of individual isoacceptor tRNA (see below and Grosjean and Westhof 2016). The knowledge of tRNA genes and copy numbers does not allow yet to predict which identified genes correspond to lowly or highly expressed cellular tRNAs (minor/major species). The low abundance of certain cellular tRNAs can be below the detection mapping method, which explains why some tRNA isoacceptors are missing in our analyses of bulk tRNAs. However, for each archaeon, representative tRNAs corresponding to each of the 20 amino acids could be analyzed. They probably correspond to the most abundant naturally occurring species. Lastly, in *Archaea*, especially the hyperthermophilic ones, tRNA genes often contain introns (Sugahara et al. 2008). Among the three archaeal species studied, the *S. acidocaldarius* contains the highest number of tRNAs with introns (21 out of 46) (Chan and Lowe 2009, 2016). Such intron-containing tRNAs are often the targets of site-specific 2′-O-ribose methylations or uridine isomerization into pseudouridine via the sRNA-guided FlpA C/D box or the Cbf5 H/ACA enzymatic machinery, respectively (see below).

### Purification and sequencing of isolated tRNA species

Each tRNA isoacceptor was isolated using two-dimensional PAGE. The process leads to a series of tRNA spots. Each spot contains generally one isoacceptor, while some contain two or rarely three isoacceptors (Supplemental Fig. S1). For each spot, all CID spectra were manually inspected and sequenced. The tRNAs were identified by unique sequences in RNase digestion products (see Materials and Methods). From such 2D-gel electrophoresis, we could purify and analyze 27 post-transcriptionally matured tRNAs corresponding to obviously major naturally occurring species from *M. maripaludis* and 30 tRNAs from *P. furiosus* (over 37 and 46, respectively) but only 22 (over 46) from *S. acidocaldarius*. In this latter case, of the 21 intron-containing primary transcripts predicted from the genome sequence only six were identified as matured species in the purified bulk matured tRNAs, while 16 over 25 of the predicted intron-less tRNAs were detected. In *P. furiosus* and *M. maripaludis*, only two tRNAs (specific for Met [CAU] and Trp[CCA]) are transcribed as intron-containing species and each of them was obtained and analyzed as the matured species.

A critical point in the present experimental strategy is to assign a modified position to the tRNA it belongs to. Indeed, because archaeal tRNAs are highly GC-rich (especially in stems), there is a high level of similarities between tRNA sequences especially for the highly conserved D- and TΨC-loops. To be sure to assign unambiguously an MS/MS sequencing spectrum, each tRNA isoacceptor was analyzed separately (see Materials and Methods). All CID (Collision Induced Decay) MS/MS spectra were manually examined and sequenced. Although LC MS/MS spectra of digestion products allow the localization of a modified nucleotide within the sequence, some of the modifications unfortunately share the same (m/z) mass. For example, methylation can be detected but, solely on the basis of the mass spectra, it is not possible to localize the methyl group (either on the ribose or on the base). The same situation occurs between uridine and pseudouridine. However, knowing the presence of a given modified nucleotide at the same position in a homogous tRNA of a closely related archaeon, together with the existence of corresponding modification enzymes (and its ORF in the genome), sometimes allows to assign the most probable chemical modification after verification that a homolog gene exists in the genome of the archaea studied (Supplemental Table S2). The ambiguous cases that are left are discussed in the text or legends. Analyses of total modified nucleosides of bulk tRNAs were also performed allowing to identify the presence of modified nucleotides that escape the above analysis of sufficiently long oligonucleotides (Supplemental Figs. S2, S3). The code for modified nucleosides used throughout this paper, except when specifically mentioned, follows either the one used in MODOMICS (Boccaletto et al. 2018) or the chemically based nomenclature (Motorin and Helm 2011; Helm and Alfonso 2014).

### Structural tRNA alignments

**FIGURE 1.**
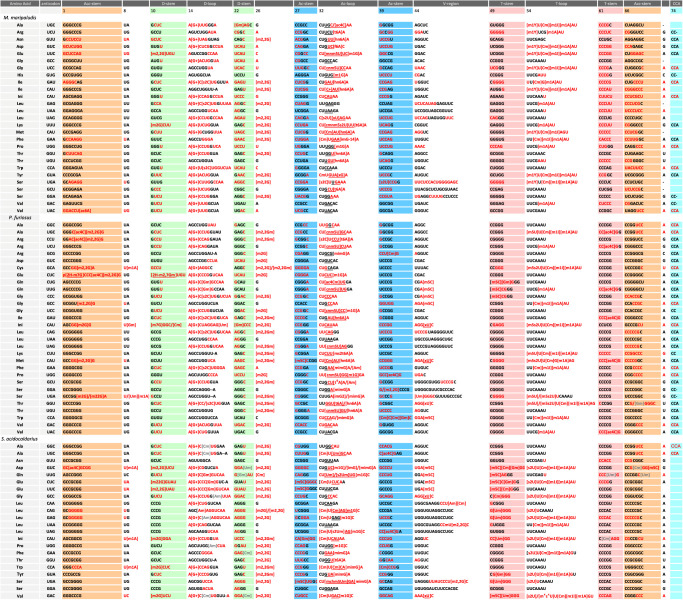
Compilation of modified tRNA sequences from *S. acidocaldarius*, *M. maripaludis*, and *P. furiosus*. Red nucleotides indicate fragments obtained by RNase T1, and/or RNase A, and/or RNase U2 digestion, while black nucleotides represent regions that could not be analyzed. Nucleotides in gray are modified nucleotides with a mass corresponding to several possible modifications.

Alignments of the sequenced archaeal tRNAs studied in this work, including the modified nucleotides we have been able to detect at the oligonucleotide digest products, are shown in Figure 1 (see also Table 1; Supplemental Fig. S10). These alignments follow the usual nomenclature of tRNA structure, and they are structural in the sense that equivalent positions in the three-dimensional structure (assumed by homology with known crystal structures) are vertically aligned. They show that the tRNA sequences follow the expected patterns of covariations and conservations as observed in the majority of cellular tRNAs of all three domains of life, demonstrating that they do conform to the known three-dimensional structure of tRNAs. The numbers of Watson–Crick base pairs (often GC-rich and with a small number of GU pairs, especially in the thermophiles) in the stems are as usual: seven in the acceptor stem (AA-stem), four in the dihydrouridine stem (D-stem), five in the anticodon stem (AC-stem) and the thymine stem (TΨC-stem). Also, a long variable region is always present in the long-arm tRNAs specific for leucine and serine. The tRNA-Leu (anticodons YAG, Y = U/C) in *M. maripaludis* and *P. furiosus* both contain, after the conserved U8, U9 instead of the very common R9 (R = A/G). This is also the case in *Thermoplasma acidophilum*, a thermo-acidic Euryarchaeon where s^4^U8 and s^4^U9 have been detected (Tomikawa et al. 2013). This occurrence of two consecutive Us at positions 8 and 9 is surprisingly accompanied by an unusual G38 opposite to U32 in the anticodon loop (especially with a CAG anticodon). Although we could not detect s^4^U at either position 8 or 9, the nucleoside analysis (Supplemental Figs. S2, S3) confirms the presence of s^4^U in both *M. maripaludis* and *P. furiosus*. An analysis of the GtRNAdb shows that these correlations stand out in *Euryarchaeota* and *Thaumarchaeota* (Chan and Lowe 2009, 2016). The D-stem presents also some particularities like a preference for G10-Y25 and Y13-G22 with the central two base pairs maintained as Watson–Crick. It is likely that some, if not all, of the uridines at positions 13, 22, and 25 of the D-stem and position 39 of the anticodon stem (AC-stem) are pseudouridines (Ψ) which cannot be differentiated from U by mass spectrometry. Moreover, the AC-stem has a pronounced preference for a G30–C40 pair, while in other tRNAs, especially of mesophilic organisms, a A30–U40 pair is present (Marck and Grosjean 2002). There are seven residues in the AC- and T-loops with the conserved residues U33 and the favored C32/A38 opposition in the anticodon loop. In the Sulfolobales clade of the *Crenarchaeota*, the tRNA-Cys(GCA) has an unusual C33 (together with G27oU43), but unfortunately we could not isolate those tRNAs. The T-loop is closed by an invariant G53–C61 pair with always the possibility to form a U54/A58 *trans* Watson–Crick/Hoogsteen stacking against it. The *trans* Watson–Crick/Hoogsteen between U8 and A14 is always present and triple formation with A21 is possible in the large majority of sequences. Interestingly, 15–48 is always a G15–C48 pair (Supplemental Fig. S11A) that stacks with the invariant A60 (from the T-loop). In three tRNA sequences (Ile, elongator Met, and Thr) of *P. furiosus*, residue 60 is G instead of the usual U, and is preceded by A59, which forms a rare combination. C48, as expected, is often methylated on C5 (m^5^C); in which case, the methyl group would be in the tRNA core and in the neighborhood of the charged formamidino group of G15 (G^+^). The positive charge on G15 points in a cavity surrounded by three negative phosphate groups from residues 7, 14, and 15. Altogether, these peculiarities correspond to tRNAs well stabilized, even in the mesophilic *M. maripaludis*.

**TABLE 1.**
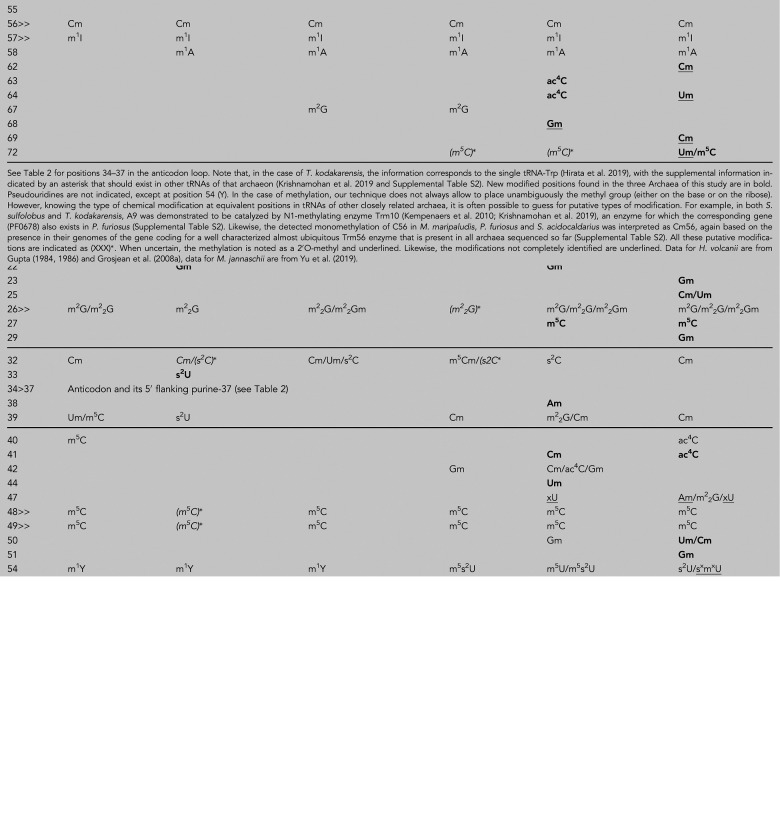
List of modified positions in different Archaea

### New modified positions

Table 1, Figure 1, Supplemental Figure S10, and the corresponding Supplemental Table S2 list all the modified nucleotides we have detected, among them several are present at newly identified positions (mostly methylations and acetylations in the two thermophilic archaea). In eukaryotic tRNAs, the m^2^_2_G modification is exclusively found at position 26 (exceptionally at position 27) at the interface between the D- and AC-stems (Machnicka et al. 2014), while in archaeal tRNAs, it is present at many other locations. The m^2^_2_G10 is observed when residue 25 is a U, in which case the m^2^_2_G modification stabilizes the G10oU25 wobble pair (discussed in Urbonavicius et al. 2006). Similarly, the 26-44 pair displays a frequent G26 modification (m^2^G, m^2^_2_G but also m^2^_2_Gm), forming either G26oU44 or G26–A44 pairs (Supplemental Fig. S11D), rarely G26–C44 (with a slight bias toward m^2^_2_G at position 26 when pairing with U44, especially for the long-arm tRNAs Leu and Ser). Surprisingly, in tRNA-Ser(GGA) of *P. furiosu*s, MS/MS data and T1 cleavage analysis (Supplemental Table S1) indicate the presence of m^2^_2_G at positions 39, which would induce a pronounced propeller-twist of the C31 = m^2^_2_G39 pair (as seen in Supplemental Fig. S11D). A similar situation exists for m^2^_2_G6–C67 in the amino acid stem. Only thermophilic archaea present the triple methylation (m^2^_2_Gm) on both the base and the ribose. Such “doubly” modified nucleosides (xNm) are hallmarks of hyperthermophilic tRNAs (McCloskey et al. 2001; Grosjean et al. 2008b; Hori et al. 2018).

In tRNAs of both Bacteria and Eukarya, the positively charged m^7^G is often found at position 46 in the variable region (Machnicka et al. 2014). m^7^G has been identified in unfractionated bulk tRNAs of two thermophilic archaea, *Thermoproteus neutrophilium* and *Thermoplasma acidophilium* (Edmonds et al. 1991). Recently m^7^G was located at position 49 of *T. acidophilum* tRNA-Leu (UAG) (Tomikawa et al. 2013). Here, we report the presence of m^7^G at position 10 of tRNA-Met^i^ (CAU) of *P. furiosus*. We suggest also the presence of a reduced neutral form of 2H-m^7^G at positions 1 and 10 of tRNA-Glu (CUC) (for position 10 we suggest an unexpected and unique case of tri-methylated 2H-m^2^_7_Gm). These suggestions are based on the following observations. The monomethylated nucleotide at position 10 of tRNA-Met^i^ has a mass of 359 Da with a neutral loss of 165 Da that is characteristic of a normal m^7^G, while at position 1 of tRNA-Glu, the methylated nucleotide has a mass of 361 Da (Supplemental Fig. S4), which could correspond to the reduced form of m^7^G (2H-m^7^G) (Supplemental Fig. S4D; Wintermeyer and Zachau 1975). The same reduced form exists for 2H-m^2^_7_Gm at position 10 of tRNA-Glu. The accuracy is less than 0.05 Da; however, in the absence of standard substance to compare against, these suggestions are still tentative. The presence of an m^7^G derivative at position 1 of an archaeal tRNA would indeed be remarkable. Usually, such guanosine derivatives are found in the form of a positively charged cap-like structure protecting the tRNA against 5′-exonucleolytic degradation (see for example Ohira and Suzuki 2016). In m^7^G cap, the 5′ extremity is a 5′OH, while our MS/MS data show a classical 5′P extremity on the *P. furiosus* tRNA-Glu (see Supplemental Fig. S4B, which corresponds to the MS/MS spectrum of the 5′-end of tRNA-Glu where, in the ion series c, c1 corresponds to p2H-m^7^G). A putative protecting role of such terminal m^7^Gp against specific exonucleolytic degradation processes remains to be demonstrated in Archaea.

Very recently, Sas-Chen et al. (2020) published a thorough analysis of ac^4^C modifications in rRNAs and tRNAs across phylogeny including several archaeal species. They found high concentrations of ac^4^C in the hyperthermophiles *Thermococcus kodakarensis, P. furiosus*, *Thermococcus sp.* AM4, and *S. solfataricus*, with a preference for CCG sites, the modified C being the middle C. Residues ac^4^C most certainly contribute to the stabilization of base pairs (Kawai et al. 1992). In this work we identified 13 ac^4^C modifications in the isolated tRNAs (Table 1; Fig. 1) and all, except the one at the wobble position 34 of tRNA-Gln (ac^4^CmUG—see below), are found in the middle position of a CCG motif within a stem. For example, in *S. acidocaldarius*, ac^4^C is found at position 41 of the AC-stem of tRNA-Leu(UAG) and at position 40 of tRNA-Ala(UGC). m5C40 is found in tRNA-Ile of *H. volcanii* (Grosjean et al. 2008a). In position 40, the modification cannot be a ribose methylation (Cm40) because the O2′(C40) is functionally engaged in H-bonds with the conserved A1339 of the 16S rRNA during the P state of translation (Selmer et al. 2006; Watson et al. 2020). Indeed, the base pairs of the AC-stem 30–40 and 29–41 are both involved in contacts with, respectively, G1338 and A1339 of the 16S rRNA in the P state (Supplemental Fig. S12A,B). In tRNA-Met^i^(CAU) of *S. acidocaldarius,* residue G29 pairing with C41 is methylated but in this case the 2′-hydroxyl group is far enough from G1338 and therefore can accommodate a methyl group, leading to a final assignment of 2′O-methyl G29 (Gm). In *S. acidocaldarius*, we found a m5C residue at position 72 in the acceptor stem of tRNA-Asp(GUC), while at the same position in tRNA-Gln(UUG), an undetermined monomethylated U72 (indicated as Um) was found. Residue m5C72 has been already reported in *S. solfataricus* tRNA-Glu/-Gly/-Met (Wagner et al. 2004), as well as in tRNA-Cys and tRNA-Thr of humans (Haag et al. 2015). Residue m5C is quite common at positions 40 (see above), 48, 49 of tRNAs in both Eukarya and Archaea (Machnicka et al. 2014). Worth to note is that, in tRNAs of *S. acidocaldarius* (and to a lower extent of *P. furiosus),* many residues are monomethylated all over the molecules. Some are catalyzed by site-specific protein-only methyltransferases while others are catalyzed by Fibrillarin-C/D box sRNP guide machinery acting on specific intron-containing pre-tRNAs (Gaspin et al. 2000; Omer et al. 2000; Clouet d'Orval et al. 2001; Dennis et al. 2001). From the database maintained by Todd Lowe (provided at http://lowelab.ucsc.edu/snoRNAdb/), this enzymatic machinery in *S. acidocaldarius* could target site-specific methylations to tRNA-Gly(CCC) at C50 in the D-arm (Tang et al. 2005; Zago et al. 2005) and to tRNA-Gln(UUG) at U34 and G18 of the AC- and D-loops, respectively (see also Ziesche et al. 2004), a situation indeed observed in the present study. However, in *S. acidocaldarius*, a few other observed monomethylated nucleotides remain orphan and await corresponding snoRNAs or stand-alone specific methyltransferases to be identified (Table 1; Supplemental Table S2, they are indicated as Xm). Likewise, in tRNAs of *P. furiosus*, several positions were predicted to be methylated via the Fibrillarin-C/D box machinery: tRNA-Trp(CCA) at C32, C34, C39, tRNA-Leu(UAA) at G47, tRNA-Leu(CAA) at C34, tRNA-Gln at C16, tRNA-Val and tRNA-Gly at G26 and tRNA-Asp(GUC) at U35 (http://lowelab.ucsc.edu/snoRNAdb/). However, although we do observe Cm34, Am38, and m^2^_2_Gm26 in some tRNAs*,* the other putative methylated positions listed above were presently not detected in the set of tRNAs we analyzed.

In *M. maripaludis*, most modified nucleotides were expected, except a methylated G22 (Gm) in tRNA-Ala(UGC) and an unidentified adenine derivative in the acceptor stem of tRNA-Val(GAC). The corresponding nucleoside A*7 displays a mass of 309.1 Da corresponding to either hypermodified m^6^_2_Am or monomodified N6-acetylA. We tend to favor the latter (ac^6^A), since such acetylated adenosine has been discovered in another methanogen (Sauerwald et al. 2005) with its location, however, tentatively assigned to residue 37. In *M. maripaludis*, a mass corresponding to f^6^A37 was found instead in tRNA-Asp (GUC) (see below). Intriguingly, in tRNA-Leu(UAG), the C32 is unmodified but U33 of the AC-loop, a very rarely modified conserved residue, is thiolated (s^2^U) (Supplemental Fig. S6). An s^2^U33 has already been found in trypanosomid mitochondrial tRNA-Trp(CCA) (Crain et al. 2002), where the stop codon is translated as Trp (Alfonso et al. 1999). The probability to find site-specific 2′O-methylations guided by snoRNA in *M. maripaludis*, is meager as the frequency of occurrence of potential intron-containing tRNA targets in mesophilic archaea is much lower than in the hyperthermophilic archaea (Sugahara et al. 2008).

### Modifications in D-loops

In mesophilic bacterial and eukaryotic tRNAs, U17 as well as a few other Us at positions 16, 20, and 20a of the D-loop, are usually modified to dihydrouridine (D). In thermophilic bacteria and hyperthermophilic archaea, this thermolabile dihydrouridine D, is rare or even totally absent (Edmonds et al. 1991), while in psychrophilic bacteria D is abundant (Dalluge et al. 1997). From these observations, Dalluge and coworkers (1977) suggested an interesting functional role for D in the maintenance of a certain degree of conformational flexibility in tRNAs, especially important to organisms growing at low temperatures where the dynamics of thermal motions of tRNAs are severely compromised. Unexpectedly, in tRNAs of *M. maripaludis* and of *P. furiosus* we found 2-thiocytidine at position 17 (Table 1; Fig. 1), a situation found also in the hyperthermophilic tRNA-Trp of *T. kodakarensis* (Hirata et al. 2019). However, in *S. acidocaldarius,* position 17 contains instead a methyl group on either C or A, most probably a 2′-O-methyl (discussed above) (Table 1). Usually, s^2^C and Cm are found at position 32 of anticodon loops (Jühling et al. 2009; Boccaletto et al. 2018). Thiolation of pyrimidines and methylation of 2′-O-ribose of nucleotides are known to favor stacking, thus limiting local flexibility of the RNA (Plesiewicz et al. 1976; Larsen et al. 2015), a property that is obviously important in organisms thriving at high temperatures.

In most crystal structures, residue 17 bulges out of the tRNA core structure and is exposed to solvent. In addition, residues 16 and 17 are in the vicinity of two other bulging residues from the T-loop, 59 and 60, and modifications in the D-loop may limit the tendency of residues 16 or 17 to bulge out of the loop. The locations of residues 16 or 17, either within or outside the tRNA core, may influence interactions between other residues in the T-D environment. When residue 16 is a pyrimidine, it is often observed that it forms a pair with residue 59, especially when 59 is also a pyrimidine. However, in the present archaeal tRNAs, residue 59 is always A and residue 60 is mostly U. In that case, a couple of crystal structures show A59 stacked on G15–C48 and U60 forming an H-bond between N3(U60) and the phosphate group between the two invariant G18–G19 of the D-loop (see for example PDB entry 2DU3, Supplemental Fig. S12C; Fukunaga and Yokoyama 2007). As stated above the modified G bearing a positively charged formamidino group (G^+^, archaeosine), a hallmark of archaeal tRNAs, also contributes to the global stability of the 3D-core of tRNA. In Bacteria and Eukarya, nucleotide 15 is never modified.

### Modifications in TΨC-loops

Nucleotide U54 is nearly always modified to thymine or 5-methyluridine (m^5^U) in Eukaryotes and Bacteria (Machnicka et al. 2014). However, in most Archaea (mainly Euryarchaeota), a 1-methylpseudouridine (m^1^Ψ) is generally found instead (Pang et al. 1982; Gupta 1984; McCloskey et al. 2001; Chatterjee et al. 2012; Yu et al. 2019). Such a modification adds a methyl group to the pseudouridine (Ψ) at the free N1 atom, a position structurally equivalent to the C5 atom of T54. In the case of *Ignococcus hospitalis*, a crenarchaeon thriving at temperatures up to 100°C, m^1^Ψ54 was shown to be further hypermodified into s^4^m^1^Ψ (Rose et al. 2020). The sulfur atom at position 4 in Ψ54 is structurally equivalent to the sulfur atom at position 2 of U54 (see Fig. 6 of Rose et al. 2020). In a few other archaea belonging to the thermococcales clade among the Euryarchaeota, as *P. abyssi*, *P. furiosus*, and *T. kodakarensis*, the bacterial-like m^5^s^2^U has been identified instead (Kowalak et al. 1994; Urbonavicius et al. 2008; Hirata et al. 2019; for review, see Hori et al. 2018).

In this work, we confirm the presence of m^1^Ψ54 in the mesophilic *M. maripaludis*, and in the hyperthermophilic *P. furiosus* a mix of m^5^U54 and m^5^s^2^U54 (Supplemental Figs. S2 and S9). For *P. furiosus*, the result is consistent with the fact that the 2-thiolation process occurs after 5-methylation of U54 and 1-methylation of A58 (Shigi et al. 2006). For *S. acidocaldarius*, the MS/MS sequencing spectra show the presence of thiolated U/Ψ (Supplemental Fig. S9A) and, only in tRNA-Val, the presence of a methylthiolated U/Ψ (Supplemental Fig. S9B). In the latter case a neutral loss of 142 was observed, which corresponds to a modified U with a methyl group and a sulfur atom, a situation that was not observed in the case of m^5^s^2^U54-containing tRNAs of *P. furiosus*. The total absence of m^5^s^2^U is also evident in the chromatogram profile of nucleoside digests from *S. acidocaldarius*, in comparison to *P. furiosus* (Supplemental Fig. S2). Altogether, these facts strongly suggest the presence of a methylthiolated derivative like s^4^m^1^Ψ as in *I. hospitalis* (Rose et al. 2020). This situation is however perplexing. Indeed, examination of the *S. acidocaldarius* genome reveals the lack of genes coding for both Pus10 (catalyzing formation of Ψ55 and Ψ54) and TrmY (catalyzing m^1^Ψ), while genes coding for TtuA and TtuB responsible for thiolation of U/Ψ (into s^2^U or s^4^Ψ) are present (Supplemental Table S2). It might be that in *S. acidocaldarius* a new type of s^2^U54/or s^4^Ψ54-methylating enzyme exists. Therefore, we prefer to indicate that position as s^x^m^x^U/Ψ54.

In all three archaea analyzed, A58 is methylated at position N1 (m^1^A58), adding a positive charge on the base while still allowing for the formation of the usual *trans* Watson–Crick/Hoogsteen 54–58 pair (Supplemental Fig. S11B). In sum, thiolation of m^5^U, m^1^Ψ and U/Um at position 54 of tRNA appears as a hallmark of (hyper)thermophilic archaea. In thermophilic bacteria, such as *Thermus thermophilus*, the thiolation process was demonstrated to be thermo-inducible, as the level of 2-thiolation of m^5^U54 increases with the cultivation temperature (Shigi et al. 2006). The van der Waals radius of the sulfur atom is 0.3 Å larger than that of the oxygen atom and its presence may fill the cavity present around that position in the overall compact T-loop, thereby excluding solvent molecules and promoting stacking.

Residue 56 is a conserved C, usually 2′-O-methylated on its ribose in almost all archaeal tRNAs analyzed so far (Clouet-d'Orval et al. 2005; Renalier et al. 2005). The ribose of C56 is highly accessible in the turn of the TΨC-loop and methylation allows protection against hydrolysis, especially at high temperatures. Cm56 forms a Watson–Crick pair with the conserved G19 of the D-loop (G19–C56) and therefore cross-bridges the two parts of the tRNA core. Residue 57 is either G or A where A57 is often doubly modified first into m^1^A, positively charged, and then into m^1^I neutral (Grosjean et al. 1995). Residue 57 intercalates between the invariant G18 and the conserved G19–C56 pair. With G57, there is an H-bond between N2(G57) or N1(G57) and one anionic oxygen atom of the phosphodiester bond between 18 and 19 and with m^1^I57 the methyl group will be on the solvent exposed surface of the T-loop. It is noteworthy that nucleotide 17 is probably 2′-O-methylated on the ribose in *S. acidocaldarius* and *P. furiosus*, and 2-thiolated in *P. furiosus* and *M. maripaludis* (Fig. 3). The 2′-O-methyl group of Cm56 could protect the bent backbone from cleavage. Both Cm56 and m^1^I57 are unique and frequent in archaeal tRNAs (Table 1), while m^1^A58 is also present in many bacterial and eukaryotic tRNAs (Jühling et al. 2009).

### Modifications at position 34 (Table 2)

The majority of the modifications observed at position 34 are the same as those identified in several tRNAs, mostly from Bacteria, such as cmnm^5^s^2^U, mnm^5^U, cnm^5^U, mchm^5^U, Cm, Um, ac^4^C, except that, in the present thermophilic archaeal tRNAs, some of them are doubly modified with an extra 2′-O-methylribose, mchm^5^Um, ac^4^Cm, s^2^Um (Grosjean et al. 2008a,b, 2010; Jühling et al. 2009). In tRNA-Met^i^ of *M. maripaludis* and *P. furiosus* (recognizable by the invariant last three G = C pairs of the AC-stem [Kuchino et al. 1982]) and tRNA-Trp of *S. acidocaldarius*, there is a modified Cm34 (Table 2). The methyl group in that position occupies a tight space in the decoding site locking C34 for pairing only with G3 (Met codon AUG or Trp codon UGG) and preventing mispairing of the C34 residue with A3 (Ile codon AUA or stop codon UGA). The Cm34 modified nucleotide found in tRNA-Trp in *S. acidocaldarius* was previously observed in *H. volcanii* (Gupta 1984) and in *T. kodakarensis* (Hirata et al. 2019). In tRNA-Ile, the anticodon CAU has to read exclusively the Ile codon AUA and the C34-tRNA is modified into agmatidine (C^+^) (Ikeuchi et al. 2010; Mandal et al. 2010). The corresponding mass spectrum for tRNA-Ile from *M. maripaludis* is shown on Supplemental Figure S8. In tRNA-Gln(CUG) of *P. furiosus*, the modified residue ac^4^Cm34, the same as in the homolog tRNA-Gln of *H. volcanii*, was found (Gupta 1984). NMR studies have shown that ac^4^Cm is exceptionally rigid in conformation owing to the additive nature of the acetylation and methylation modifications which stabilize the 3′-*endo* sugar conformation (Kawai et al. 1992). The same remark probably applies to Um34 and s^2^Um34 that occur in tRNA-Gln(UUG) and tRNA-Leu(UAG) of *S. acidocaldarius*. Interestingly, ac^4^C residues were also found in the acceptor stems of *P. furiosus* and *S. acidocaldarius*, as well as in the AC-stem of *S. acidocaldarius* (see above and Table 1). Modification of U34 is necessary for decoding G3 ending codons. The modification in U34 (U34*) changes the chemical structure of the U34 so that a pair U34*–G3, with the U displaced into the minor groove, and not into the major groove, can be stabilized (Rozov et al. 2016; Westhof et al. 2019). Several U34-containing tRNAs were observed modified either at position C5 and/or at position C2. The case of xU34 in tRNA-Leu of *M. maripaludis* is explained below.

**TABLE 2.**
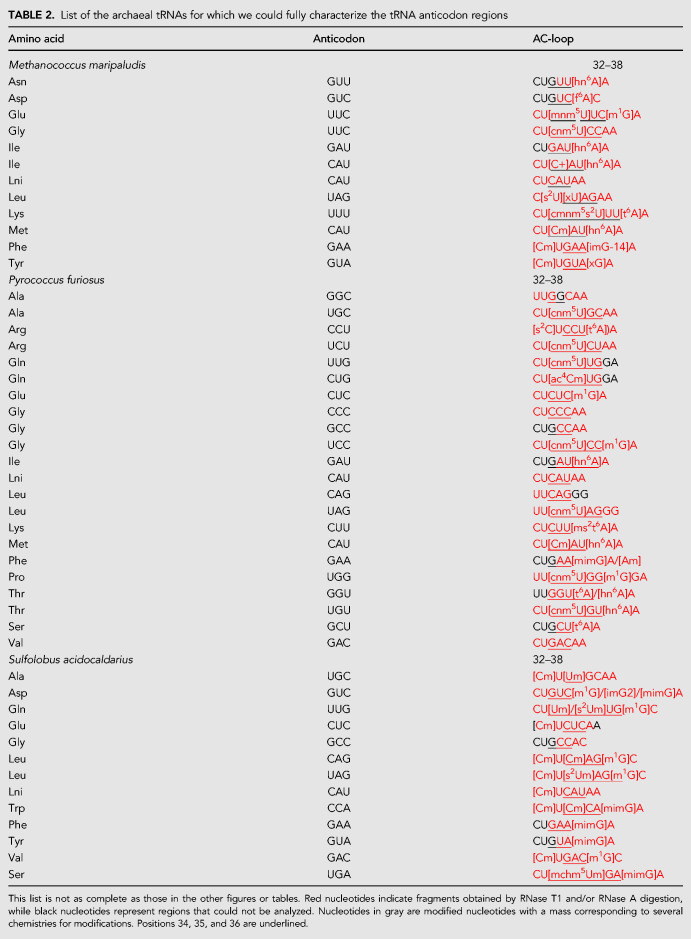
List of the archaeal tRNAs for which we could fully characterize the tRNA anticodon regions

### Wyosine and modifications at position 37

**FIGURE 2.**
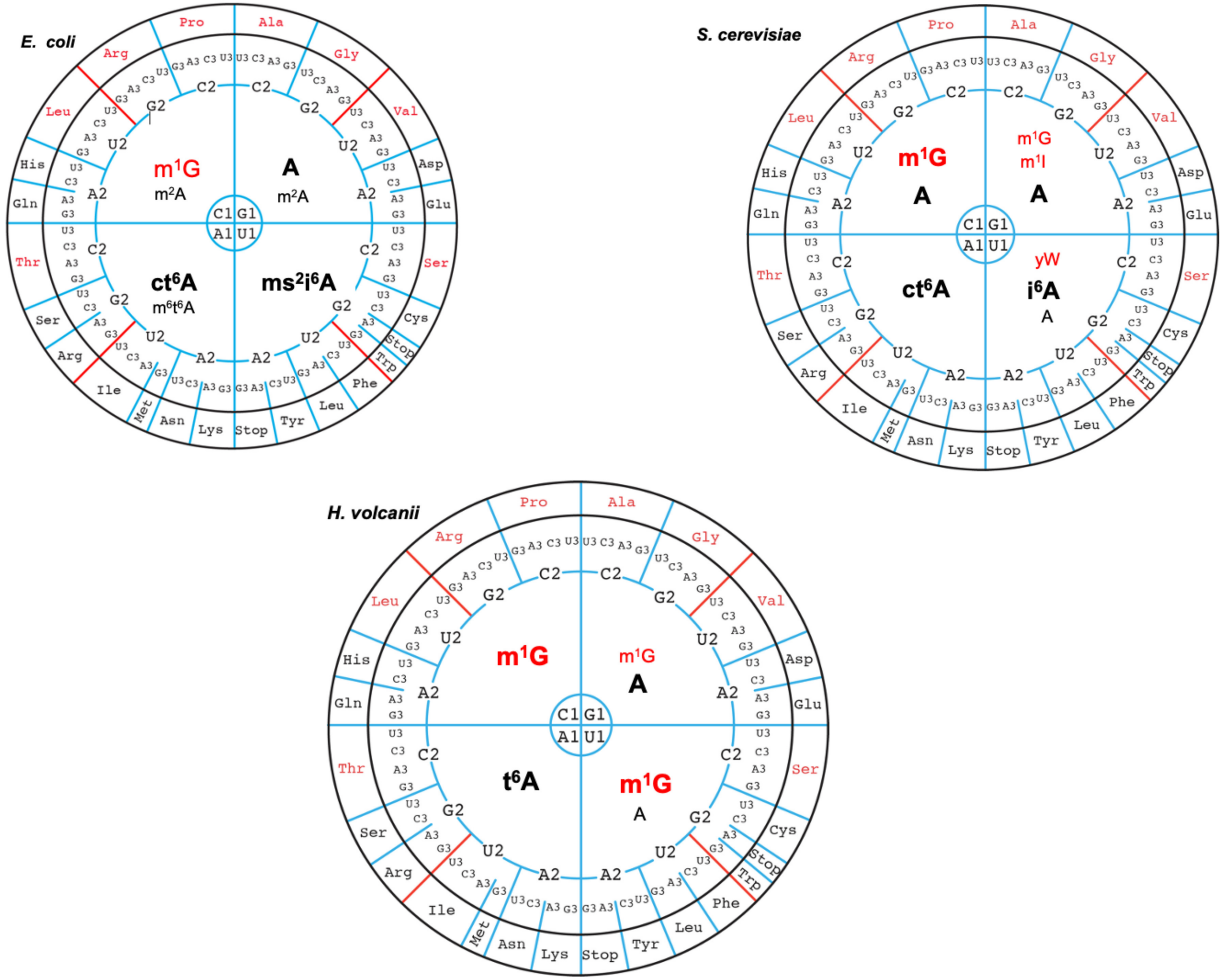

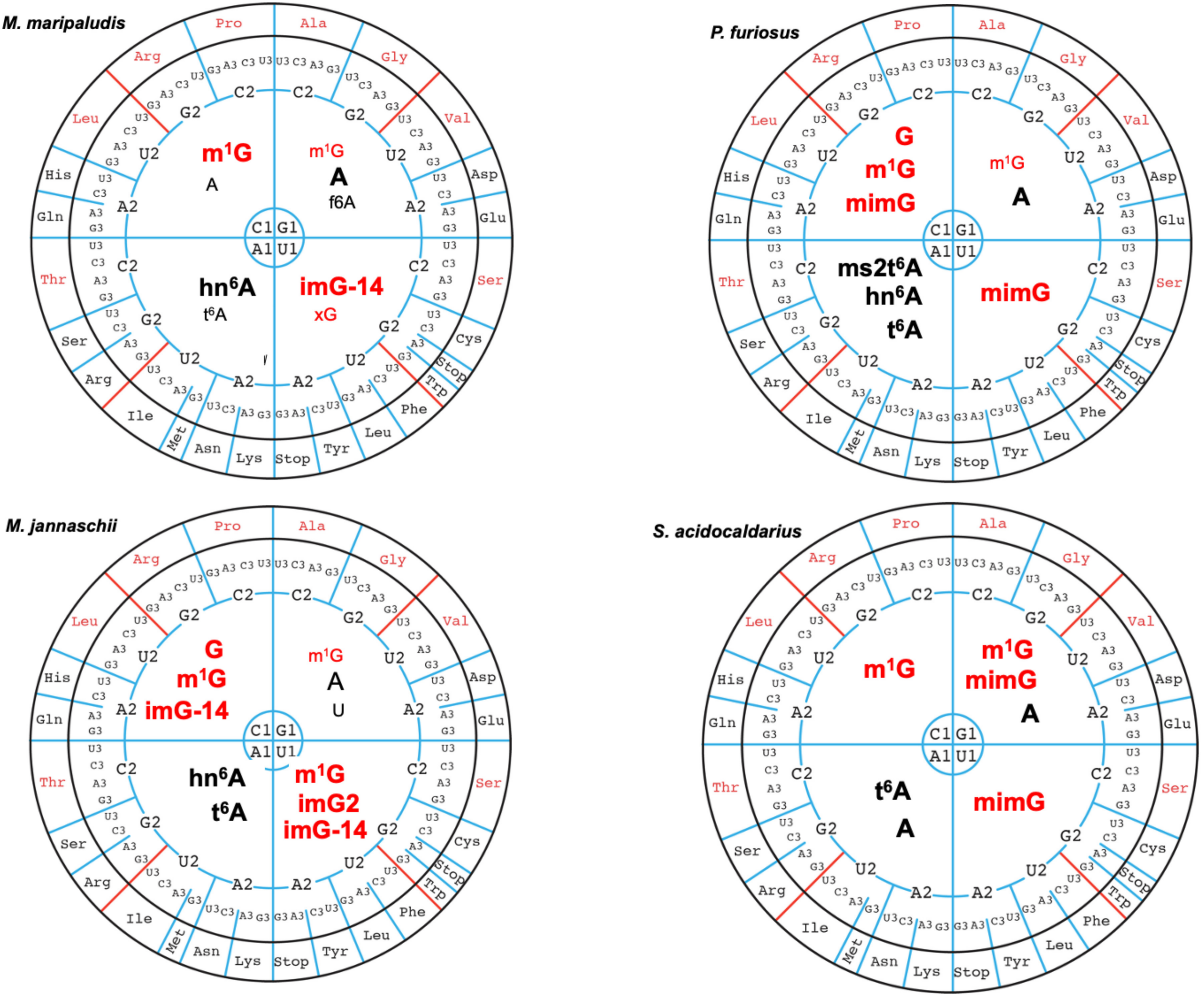
Patterns of distribution of modified nucleotides at position 37 in *E. coli*, *S. cerevisiae* and five archaeal species on the wheels of the genetic code (Grosjean and Westhof 2016). In that representation, GC-rich codons are at the *top* and AU-rich at the *bottom* of the wheel. The codon positions are numbered 1, 2, 3 so that G1A2C3 gives an Asp codon. The four quadrants are characterized by the first codon position. Data for *E. coli* and *S. cerevisiae* are from MODOMICS (Boccaletto et al. 2018). Data for *H. volcanii* are from Gupta (1984, 1986) and Grosjean et al. (2008a), data for *M. jannaschii* are from Yu et al. (2019). The wheels for *E. coli*, *S. cerevisiae*, and *H. volcanii* are adapted from Grosjean and Westhof (2016). In each quadrant, the observed modified nucleotides at position 37 are shown (red for G derivatives and black for A derivatives; the very unusual U37 of tRNA-Ala(GGC) of *M. jannaschii* is also shown in black). The modifications observed frequently have a large font and are bold, while those observed only once or in a single tRNA species have a smaller font and are not bold.

Residue 37 is commonly a purine, often (hyper)modified, that stacks on the first 1–36 bp formed between the codon and the anticodon during translation on the ribosome. Residue 37 should not be 2′-O-methylated because it forms an H-bond with N6(A1913) of helix H69 from the large subunit in the A state in known crystal structures of ribosomes (Supplemental Fig. S12D; Selmer et al. 2006). The type of modification at base 37 usually correlates with the rest of the so-called extended anticodon stem–loop, especially with the adjacent nucleotide 36 of the anticodon (Yarus 1982; Grosjean and Westhof 2016). In *E. coli*, for example, m^1^G exclusively occurs in tRNAs recognizing codons CCN (Pro), CGN (Arg), CUG (Leu), that is, in tRNAs decoding in the codon quadrant starting with C1 (Fig. 2; Supplemental Fig. S10). All the other tRNAs, belonging to the three remaining decoding quadrants, harbor either an unmodified A37 or a modified A37 (m^2^A, t^6^A, m^6^t^6^A, ms^2^i^6^A) with large modifications in the codon quadrants starting with U1 or A1 (Fig. 2; Supplemental Fig. S10). In the halophilic mesophilic *H. volcanii*, m^1^G37 occurs in tRNAs decoding codons starting with C1 (G-ending codons) and U1 (A-ending codons, with the exceptions of tRNA-Ser(CGA), tRNA-Ser(GGA) where A38 is found and tRNA-Glu(CUU) with m^1^G; Gupta 1986). In all the other tRNAs of *H. volcanii*, A37 or a modified A37 is used (Grosjean et al. 2008a).

In *M. jannaschii*, *P. furiosus, M. maripaludis*, and *S. acidocaldarius*, the landscape is striking. Indeed, the tRNAs for the codon quadrants starting with C1 and U1 contain m^1^G but also imG-14/imG2, a wyosine derivative of m^1^G37 that is reminiscent of the yW37 found exclusively in tRNA-Phe of Eukaryotes (de Crécy-Lagard et al. 2010). One finds indeed imG-14 or mimG at position 37 of tRNA-Phe(GAA) in *M. maripaludis* and *P. furiosus* with a wyosine-like (xG) (as discussed below), in tRNA-Tyr(GUA) of *M. maripaludis* and mimG37 in tRNA-Arg(GCG) of *P. furiosus* (Fig. 2; Supplemental Fig. S10). In *S. acidocaldarius*, wyosine derivatives are present in tRNA-Asp(GUC), tRNA-Ser(UGA), tRNA-Phe(GAA), tRNA-Trp(CCA), and tRNA-Tyr(GUA). This surprising result was noted in the case of *M. jannaschii* (Yu et al. 2019) where besides tRNA-Phe, the tRNA-Arg(UCG), tRNA-Cys(GCA), tRNA-Leu(UAA), tRNA-Ser(GGA), and tRNA-Tyr(GUA) contain wyosine derivatives (Fig. 2). In *T. kodakarensis* (Hirata et al. 2019), mimG was found at position 37 of tRNA-Trp. Obviously, the presence of wyosine derivatives is more prevalent in Archaea than in Eukarya, especially in the U1-quadrant.

The tRNAs corresponding to the codon quadrants starting with A1 still prefer large modifications on A37 (t^6^A, ms^2^t^6^A, hn^6^A), while the G1-quadrant prefers A and in a few isolated cases also m^1^G (Fig. 2; Supplemental Fig. S10). In the case of tRNA-Asp of *M. maripaludis*, a modified A37, which has the same nucleoside mass (295.1) than either di-methyl-A (m^6^_2_A) or N6-formyl-A (f^6^A) was found. The latter f^6^A derivative is the most probable modification. The modified f^6^A derivative has been identified in mammalian mRNAs (Fu et al. 2013). Interestingly, as discussed below, in *M. maripaludis*, a mass corresponding to ac^6^A, a modified nucleotide in the same biochemical pathway as f^6^A, was observed for residue 7 of the acceptor stem. The homologous tRNA in *M. jannaschii* harbors an unmodified A. There is also one report of the presence of m^6^_2_A in *Mycobacterium bovis*, but without identification of either tRNA species or tRNA position (Chan et al. 2011). Also, in *E. coli*, tRNA-Val(UAC) contains m^6^A37 (Golovina et al. 2009).

Thus, as a rule, in *Archaea*, tRNAs decoding the codon quadrant starting with C1 and U1 harbor either unmodified A37 or mostly modified G37. The G1-quadrant has a preference for unmodified A or slightly modified A with some occurrences of modified G37. While tRNAs decoding the codon quadrant starting with A1 seem to harbor mostly hypermodified A37. In short, the stacking power of a G37 derivative (modified or not) on the first codon–anticodon base pair (Y1-R36) is obviously preferred for efficient decoding on the ribosome, while for R1-Y36 another type of stacked hypermodified A37 is favored. In addition to residue 37, other elements of the extended anticodon stem–loop, including generally simpler chemical modifications on the base and/or the ribose also contribute to the global efficiency and accuracy of the translation process, whatever the temperature at which the archaeon is growing. These rules are more restrictive than those observed in other organisms like *E. coli, S. cerevisiae* or *H. volcanii* (Fig. 2; see Grosjean and Westhof 2016).

### Tentative identification of three novel modified nucleosides

The sequence analysis of each tRNA allowed the detection of three possibly novel chemical modifications (designated xG and xU). The first one was found at position G37 of tRNA-Tyr(GUA) of *M. maripaludis* (Supplemental Fig. S5) with a nucleoside mass of 392 Da, much higher than for the expected m^1^G37. In the homolog tRNA-Tyr of *M. jannaschii*, G37 is unexpectedly imG-14 of the wyosine metabolism (Yu et al. 2019). If this is also the case for *M. maripaludis* tRNA-Tyr, on the basis of previous work, one would therefore expect to find either imG, yW-86 or yW-72 (see G37 pathway #5 in Fig. 4 of de Crécy-Lagard et al. 2010). Taking into account that CID fragmentation of xG37 occurs between guanine and the modification, the MS/MS spectrum shows the complete mass of guanosine (345 Da) and a neutral loss of 109 Da representing the mass of the adduct (Supplemental Fig. S5). The nucleoside mass of xG (392 Da) could therefore correspond to yW-72 (436.17 Da) with the loss (natural or accidental) of the carboxyl group (44.17 Da).

The second novel modification is xU at position 34 of tRNA-Leu(xUAG) of *M. maripaludis* with a nucleoside mass of 269 Da. Again, in several tRNAs of *M. jannaschii* (Yu et al. 2019), either 5-cyanomethyl-U (cnm^5^U) or 2-thiolated-5-cyanomethyl-U (cnm^5^s^2^U) have been found, as in other archaeal tRNAs (Mandal et al. 2014). We propose that xU34 in *M. maripaludis* is the simpler 5-cyano-U (cn^5^U), with the cyano group directly linked to the C5 atom of uracil. Such a derivative is known from organic chemistry (Mao et al. 2018) but was never identified in tRNA so far.

A third unidentified modification, with a nucleoside mass of 338 Da, was found at position U47 of two tRNAs of *S. acidocaldarius,* tRNA-Val(GAC) (Supplemental Fig. S7A) and tRNA-Gly(GCC) (Supplemental Fig. S7B), two tRNAs of *S. acidocaldarius,* tRNA-Met elongator(CAU) (Supplemental Fig. S7C) and tRNA-Thr(UGU) of *P. furiosus*. In Bacteria and Eukarya, 3-(3-amino-3-carboxypropyl)-uridine (acp^3^U, nucleoside mass 345.1 Da) is widely conserved in the D- and variable loops (Takakura et al. 2019). It is likely that in hyperthermophilic archaea U47 is modified differently.

## DISCUSSION

**FIGURE 3.**
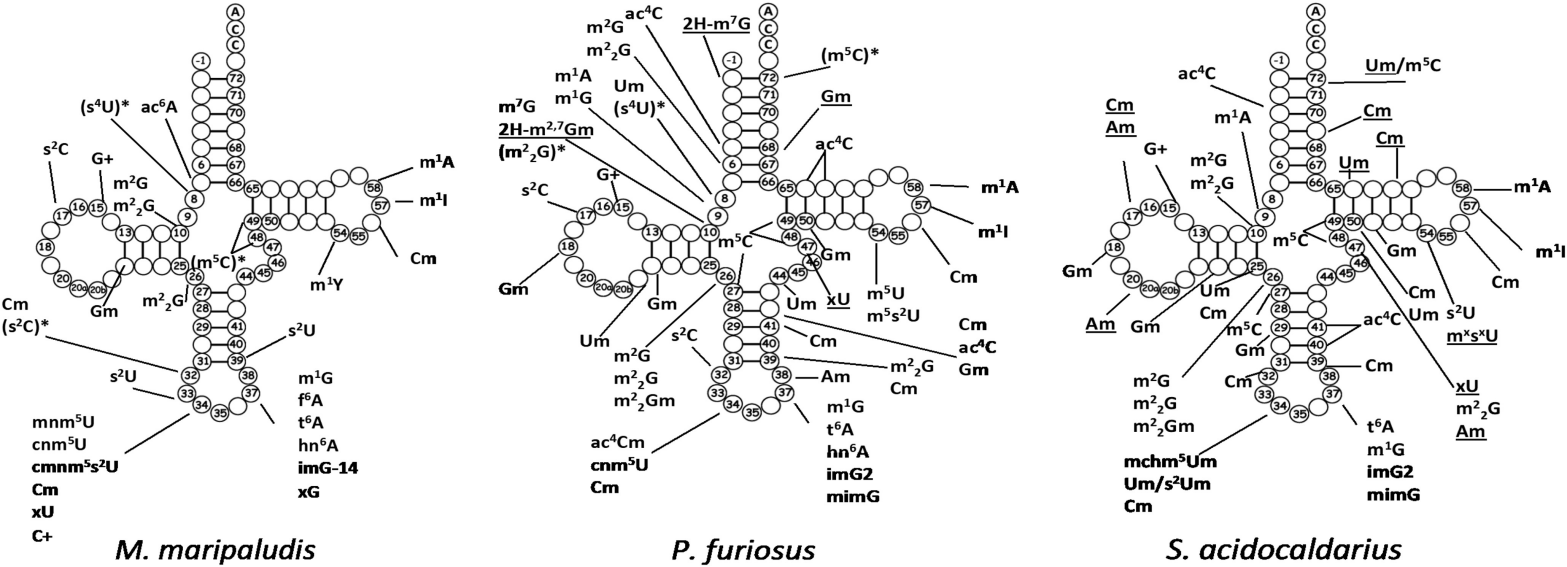
Nucleoside modification patterns in archaeal tRNAs determined by experimental MS/MS sequencing. The different modifications observed are reported on a typical cloverleaf two-dimensional structure. Underlined nucleotides are modified nucleotides with a mass corresponding to several possible chemistries for modifications, most of them corresponding to simple monomethylation products (see legend to Table 1). A “/” between two notations means a mixture of modifications. An “x” before a nucleotide, for example, xG, or after a modification symbol, for example, m^x^, means that the modification has not been formally identified (see text). Modified nucleotides indicated in brackets followed by an asterisk correspond to modifications deduced from the convergence of information from both the analysis of total tRNA and the presence of the gene coding for the corresponding modification enzyme in the genome of the particular archaeon. The modifications, uncertain or not completely identified, are underlined. Pseudouridines are not represented, except in the case of N1-methylpseudouridine (m^1^Y) because of the methyl group.

All the tRNA modifications identified in this work are compiled in several figures and tables. Figure 1 and Supplemental Figure S10 align the intron-less tRNA sequences as deduced from the genomes on which the modified nucleotides are indicated; Figure 3 summarizes the data in cloverleaf representations; Supplemental Figure S3 displays the relative amounts of modified nucleosides identified in bulk tRNAs; Supplemental Tables S2 and S3 list the oligonucleotide fragments obtained after RNase digests. Supplemental Table S2 lists all the archaeal tRNA modification enzymes (and their corresponding coding genes) that have been experimentally validated so far in independent works or deduced from their close similarities with genuine modification enzymes in each of the Methanococcales, Pyrococcales and Sulfolobales groups of Archaea. Altogether, 79 naturally occurring fully matured isoacceptor tRNAs coding for 20 amino acids have been analyzed out of a theoretical total set of 116 species. Thus, the possibility still exists that a few modifications that are specific to the missing tRNAs (especially in the anticodon loop including the wobble position 34) still escaped our analysis.

Nevertheless, the major general conclusions that came out from this comparative analysis of modified nucleotide patterns in three very different archaeal species are the following: (i) the least diversified chemical modification pattern is observed in the mesophilic *M. maripaludis*; (ii) a larger diversity of modifications is found in the two hyperthermophiles; (iii) the largest amount of 2′-hydroxyl ribose methylations occurs in the acidophilic hyperthermophile *S. acidocaldarius*, most of them appear being catalyzed by Fibrillarin-C/D box sRNP guide machinery; (iv) depending on the tRNA species, nucleotides at positions 32, 38, and 39 of the extended anticodon loop are frequently but diversely modified; (v) beyond those found at positions 34 and 37 in the anticodon loop, a few characteristic modifications are found in the body of most if not all isoacceptor species of the three archaea (G^+^15 in the D-loop, m^2^G/m^2^_2_G at positions 10 and 26 at the beginning and the end of the D-arm, m^5^C at positions 48/49 of the variable loop, Cm56, m^1^I57 and m^1^A58 in the TΨC-loop), some of them are hallmarks of archaeal tRNAs; (vi) except for ac^4^C at the wobble position 34, this modified residue is present in the amino-acceptor and anticodon stems of only thermophilic archaeal tRNAs; (vii) at variance with the situation in bacterial and eukaryal tRNAs, m^2^G/m^2^_2_G are found at positions other than 10 and 26; (viii) remarkably, the chemical adducts on the conserved U54 of the T-loop depends on archaeon analyzed (m^1^Ψ, m^5^U/m^5^s^2^U, or s^4^m^1^Ψ); (ix) the suggested presence of m^7^G modifications at the nucleotides 1 and 10 in *P. furiosus* would also be remarkable.

All these observations complete and reinforce similar conclusions made by others about the importance of certain post-transcriptional modifications for correct tRNA folding and on final cellular stability of the already G/C-rich tRNAs in thermophiles (Edmonds et al. 1991; Kowalak et al. 1994; McCloskey et al. 2001; Noon et al. 2003; for reviews, see Machnicka et al. 2014; Lorenz et al. 2017; Hori et al. 2018). As a rule, methylations promote precise H-bonded pairs (e.g., m^1^A favors Hoogsteen pairs or m^2^_2_G favors GoU or GoΨ pairs) and electrostatic charges introduced by the chemical adducts are localized in shielded pockets of the tRNA fold (m^1^A, m^7^G, archaeosine G^+^). Modifications in the tRNA core, although not close in sequence, tend to form clusters of modifications filling empty space on the surface of the compact tRNA core. In addition, methylations on the base and/or the ribose affect the hydration shells in complex ways (Auffinger and Westhof 2001). Thiolation of U or C, acetylation of C, and isomerization of U to Ψ, can stabilize the 3′-*endo* sugar conformation, fill in space and enhance stacking power or base-pairing (Kawai et al. 1992; Davis 1995; Larsen et al. 2015; Sas-Chen et al. 2020). High-resolution crystallographic structures would be necessary to apprehend the effects of such complex modification scaffolds.

## MATERIALS AND METHODS

### Culture and tRNA isolation

Total tRNA of *M. maripaludis* and *P. furiosus* were prepared as described in de Crécy-Lagard et al. (2010). Total tRNA of *S. acidocaldarius* was obtained from a 12 L culture using the procedure described in Buck et al. (1983).

### Individual tRNA purification by two-dimensional PAGE

tRNA isoacceptors were isolated using two-dimensional gel electrophoresis as previously described (Antoine et al. 2019; Antoine and Wolff 2020). Briefly, the total tRNA of each organism was separated in a first dimension gel under denaturing conditions using 12.5% polyacrylamide gel and 8 M urea, followed by a second dimension under semi-denaturing conditions using 20% polyacrylamide gel and 4 M urea at room temperature (Supplemental Fig. S1). Gel are staining with an ethidium bromide solution (10 µg.l–1) for 10 min. Spots containing tRNAs are visualized and excised under UV light (302 nm).

### In-gel RNase digestion

Gel spots containing tRNAs were dried and rehydrated by 20 µL of 0.1 U/µL of RNase T1 (ThermoFisher Scientific) or by 20 µL of 0.01 U/µL of RNase A (Thermo Fisher Scientific) in 100 mM ammonium acetate (pH is not adjusted). For a few selected samples, spots were digested by RNase U2, by using 50 µL of RNase U2 (homemade) at 0.3 ng.µL^−1^ in 100 mM ammonium acetate (pH is not adjusted). The spots were incubated 4 h at 50°C. Using ZipTip C18 (Milllipore) samples were desalted by several washes with 200 mM ammonium acetate and eluted with 50% acetonitrile in milliQ water and dried under vacuum.

### NanoLC-MS/MS of RNA oligonucleotides

Pellet containing RNase digestion products is resuspended in 3 µL of milliQ water and separated on an Acquity peptide BEH C18 column (130 Å, 1.7 µm, 75 µm × 200 mm) using a nanoAcquity system (Waters). The column was equilibrated in buffer A containing 7.5 mM TEAA (Triethylammonium acetate), 7.0 mM TEA (Triethylammonium) and 200 mM HFIP (Hexafluoroisopropanol) at a flow rate of 300 nL/min. Oligonucleotides were eluted using a gradient from 15% to 35% of buffer B (100% methanol) for 2 min followed by elution with an increase of buffer B to 50% in 20 min. MS and MS/MS analyses were performed using SYNAPT G2-S (quadrupole time-of-flight mass spectrometer) from Waters Corporation. All experiments were performed in negative mode with a capillary voltage set at 2.6 kV and a sample cone voltage set at 30 V. Source was heated to 130°C. The samples were analyzed over an *m/z* range from 500 to 1500 for the full scan, followed by fast data direct acquisition scan (Fast DDA).

### Data analysis

All CID were deconvoluted using MassLynx software from Waters and manually sequenced by following the y and/or c series (w ions were also useful when sequencing was difficult or in order to confirm the sequence). Experimental masses of parents and fragments were compared to the theoretical masses obtained by the Mongo Oligo Mass Calculator (https://mods.rna.albany.edu/masspec/Mongo-Oligo; Cantara et al. 2011). tRNA identification was done by comparisons with the genomic sequences obtained from GtRNAdb (http://gtrnadb.ucsc.edu/; Chan and Lowe 2009, 2016). Data about nucleoside modification were obtained from Modomics (Boccaletto et al. 2018).

### Data analysis by MassSpec-Toolkit for RNAs

The identification and characterization of modified tRNAs by LC-MS/MS spectrum analysis is difficult and time-consuming. To help in this process, we implemented MassSpec-Toolkit for RNAs, a Python web application (http://labex-ibmc.u-strasbg.fr/MassSpec-Toolkit/, accessible upon request) linked to a local MongoDB database that stores user-provided RNA genomic sequences and their theoretical digestion products obtained by specific ribonucleases. For each studied species, mature tRNA sequences retrieved from GtRNAdb (Chan and Lowe 2009, 2016) were submitted to the application and digested in silico with RNases T1, A and/or U2, in the “RNA Digestion” module. Genomic tRNA sequences longer than 100 nt were discarded and for the remaining ones with a nonambiguous anticodon position, fragments with common U34 or G34 and/or A37 or G37 modifications were generated when appropriate. In addition, fragment variants containing up to five additional methylations were also computed for each digestion product. Experimental data such as parent ion masses or manually reconstructed subsequences could then be compared to the theoretical ones in the “RNA Search” module. Additional criteria, like the species of interest, the ribonuclease used, as well as the presence of expected methylations or modifications at specific positions, can be specified to reduce the search space in the database. Candidate tRNAs are given a score comprised between 0 and 1 depending on the number of matching masses or subsequences they present with the list provided by the user. Besides these two main modules, the application gathers a set of “Additional Tools” under a third module that includes some of the tools present in the Mongo Oligo Mass Calculator (Cantara et al. 2011) and “Total Mass Decipherer.” The latter program can be very useful to identify modifications in case of incomplete MS/MS series, since it computes all combinations of a chosen set of (modified) nucleotides matching the mass of an RNA fragment obtained after cleavage by RNase T1.

### RNase cleavage

LC MS/MS of digestion products allows the localization of methylation in the correct nucleotide but does not allow the localization on the ribose or on the base (Supplemental Table S1). To correctly assign the type of methylation, we used known modified tRNA sequences and the presence of modification enzymes in the species genome (Supplemental Table S2). To confirm the type of methylation, we also used RNase T1 and RNase A cleavage profiles. Indeed, a methyl group on the 2′ ribose protects RNA against RNase cleavages. With the methyl group on the base, the interpretation of RNase cleavages is not straightforward. Our data show that in the case of RNase T1, m^2^G and m^2^_2_G can be cleaved (one example for each, m^2^G10 in *S. acidocaldarius* tRNA-Trp and m^2^_2_G39 in *P. furiosus* tRNA-Ser[GGA]) but m^7^G, m^1^G are never cleaved. With RNase A, m^5^C and m^1^Y are cleaved and Um, m^5^U and m^5^s^2^U are not cleaved. It is interesting to note that, for tRNA-Cys and tRNA-Met of *P. furiosus* tRNAs, RNase A does not cleave m^5^U54. A previous work shows that m^5^U54 is always cleaved (Antoine and Wolff 2020). A possible explanation is the presence of a stretch of three to four Gs 5′ preceding the modified m^5^U54 that could prevent access and binding of RNase A.

### LC-MS/MS of nucleosides

Total tRNA was desalted by ethanolic precipitation with 200 mM ammonium acetate (Supplemental Figs. S2, S3). For nucleoside analysis, tRNAs are diluted to a concentration of 5 µg/µL in H_2_O. Digestion was carried out in the following order: 14 µL H_2_O; 2 µL buffer P1 10× (2 mM ZnCl_2_, 250 mM NH_4_OAc, pH 5.0); 21 µL of tRNA and 2 µL of P1 (0.5 U/µL). The mixture is incubated at 37°C for 2 h followed by addition of 2 µL of snake venom phosphodiesterase (0.1 U/µL) for 4 h at 37°C. After digestion, 20 µL of BAP (1.5 U/µL in 100 mM NH_4_OAc) were added to the mixture. The latter was then incubated at 37°C for 2 h, dried under vacuum SpeedVac and resuspended with 100 µl of methanol. Nucleosides were analyzed by liquid chromatography coupled to mass spectrometry using an Ultimate 3000 (Thermo) chromatography coupled to an EvoQ triple quadrupole (Bruker). Separation was performed on an Acquity UPLC HSST3 column (1.8 µm, 2.1 × 100 mm, Waters) equipped with an Acquity UPLC HSST3 precolumn (1.8 µm, 2.1 × 5 mm, Waters). A gradient of solvent A (H_2_O, 0.1% formic acid [Sigma Aldrich]) and solvent B (methanol [Fisher Chemicals], 0.1% formic acid [Sigma Aldrich]) was used as follows: 2% B during 2 min, 7% B at 4 min, 100% B at 12 min, hold during 1.5 min and back to 2% of B at 13.5 min, hold during 1.5 min for a total run time of 15 min. The column was operated at 35°C with a flow rate of 0.32 mL/min; 10 µL of samples were injected for each run. The triple quadrupole was used in positive ion mode, the spray voltage was set at 3500 V and cone temperature at 350°C. Nucleosides were identified using multiple reaction monitoring (MRM) with one to three transitions per nucleotide. The identifications were based on the retention time, *m/z* of the parent ion and *m/z* of the daughter ions in MS Data Review software (Bruker), with a signal-to-noise (S/N) ratio set at 10 and a search window of ±0.2 min.

## SUPPLEMENTAL MATERIAL

Supplemental material is available for this article.

## Supplementary Material

Supplemental Material
