# Supplementary material for: Comparative patterns of modified nucleotides in individual tRNA species from a mesophilic and two thermophilic archaea
Source: RNA. 2020 Dec;26(12):1957–75. doi: 10.1261/rna.077537.120 (PMC7668247; doi:10.1261/rna.077537.120)
Supplement: Supplemental Material [file supp_26_12_1957__index.html]

Comparative patterns of modified nucleotides in individual tRNA species from a mesophilic and two thermophilic archaea — Supplemental Material 

# Comparative patterns of modified nucleotides in individual tRNA species from a mesophilic and two thermophilic archaea

## Supplemental Material

- Supplemental\_Material.pdf
