# Supplementary material for: Comparative patterns of modified nucleotides in individual tRNA species from a mesophilic and two thermophilic archaea
Source: RNA. 2020 Dec;26(12):1957–75. doi: 10.1261/rna.077537.120 (PMC7668247; doi:10.1261/rna.077537.120)
Supplement: Supplemental Material [file supp_077537.120_Supplemental_Material_.pdf]

Comparative patterns of modified nucleotides in individual tRNA species from a mesophilic and two thermophilic archaea

Philippe Wolff[1]*, Claire Villette[2], Julie Zumsteg[2], Dimitri Heintz[2], Laura Antoine[1], Béatrice Chane-Woon-Ming[1], Louis Droogmans[3], Henri Grosjean[3], Eric Westhof[1]*

[1] Architecture et Réactivité de l'ARN, Institut de Biologie Moléculaire et Cellulaire du CNRS, Université de Strasbourg, F-67084, Strasbourg, France
[2] Institut de Biologie Moléculaire des Plantes du CNRS, Université de Strasbourg, F-67084, Strasbourg, France
[3] Laboratoire de Chimie Biologique, Université Libre de Bruxelles, Institut Labiris, Avenue Emile Gryzom 1, B-1070, Belgium

SUPPLEMENTAL MATERIAL

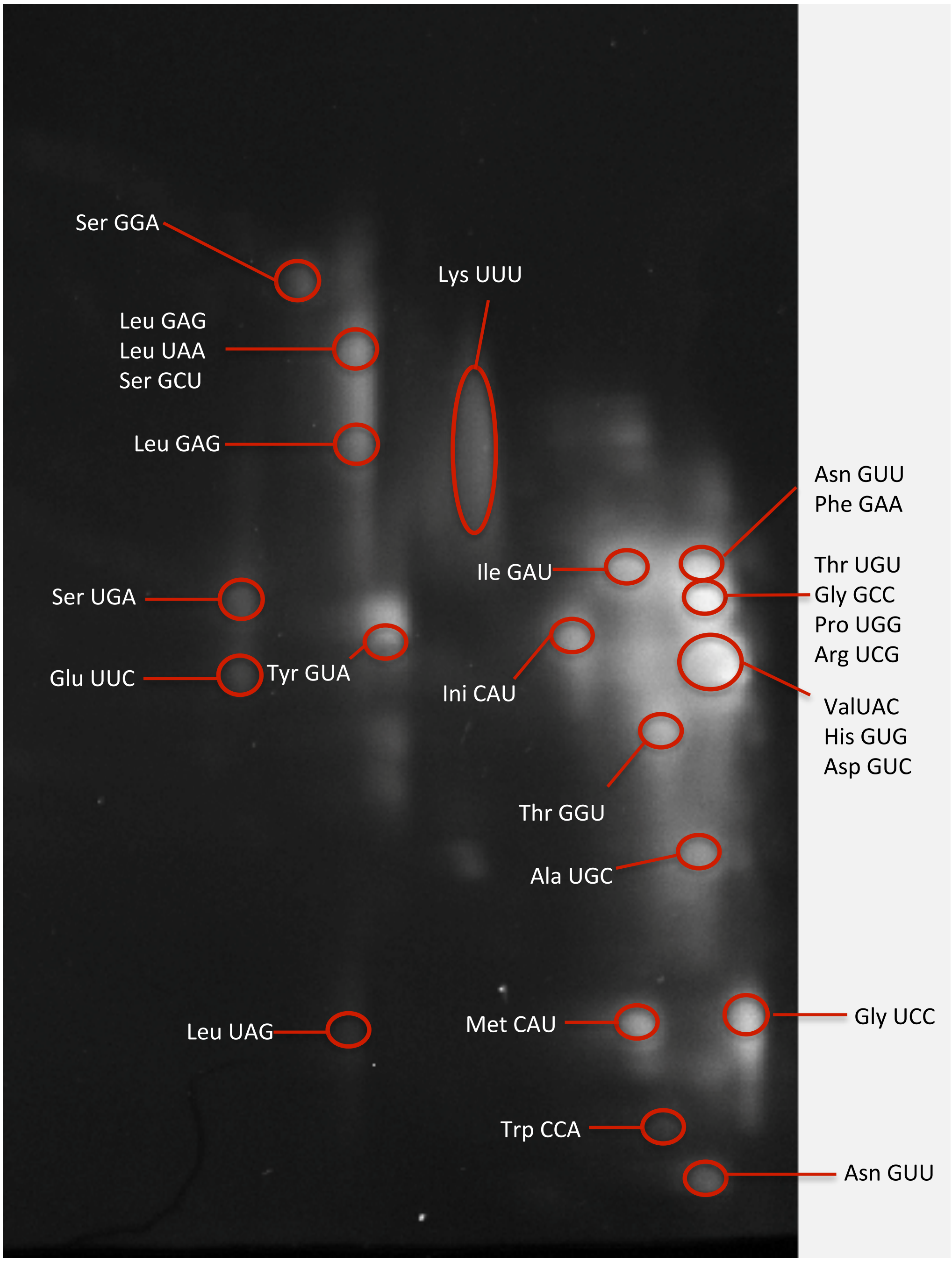
Ser GGA
Lys UUU
Leu GAG
Leu UAA
Ser GCU
Leu GAG
Asn GUU
Phe GAA
Ile GAU
Thr UGU
Gly GCC
Pro UGG
Arg UCG
Ser UGA
Tyr GUA
Glu UUC
Ini CAU
ValUAC
His GUG
Asp GUC
Thr GGU
Ala UGC
Leu UAG
Met CAU
Gly UCC
Trp CCA
Asn GUU

A

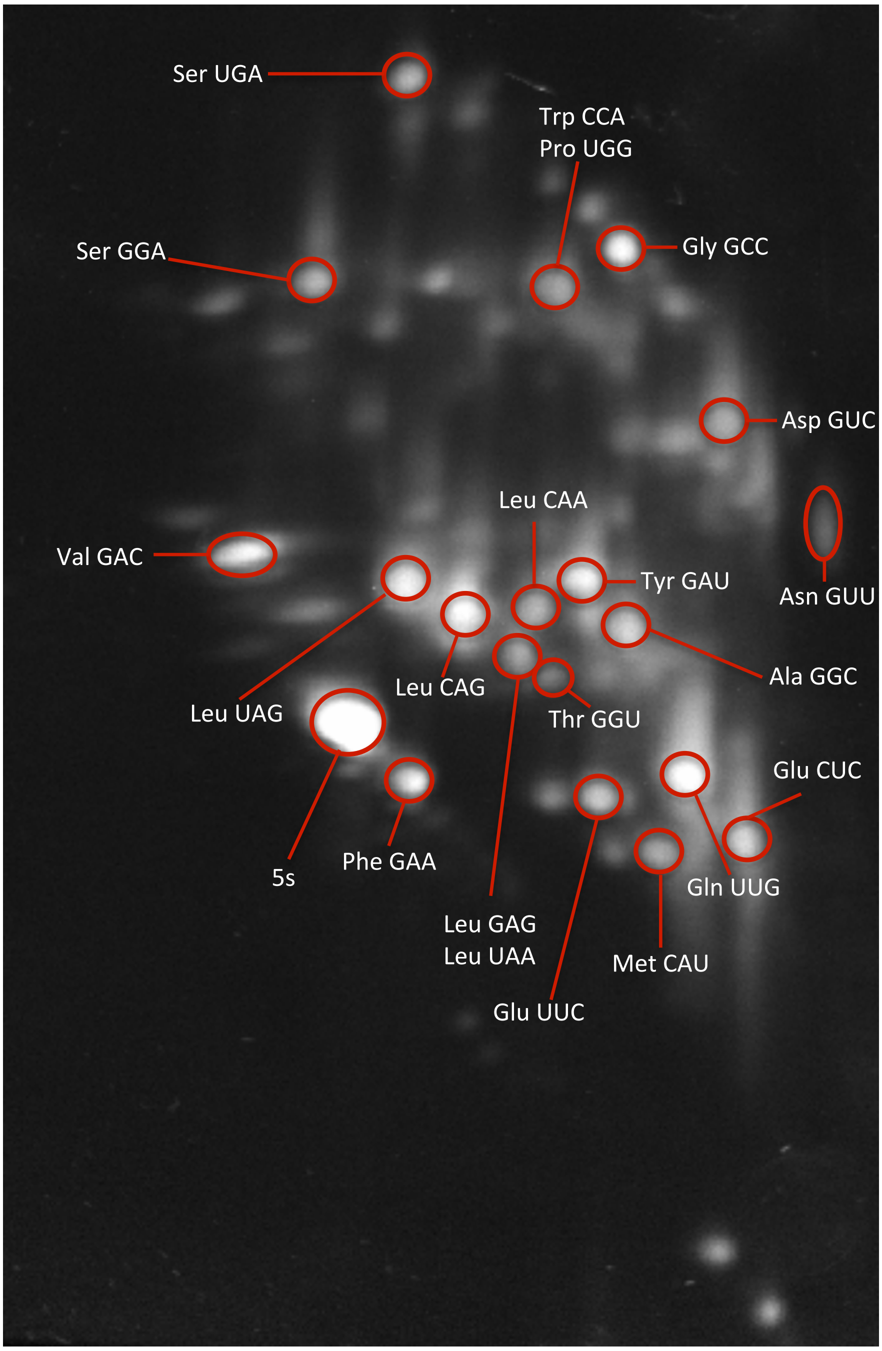
Ser UGA
Trp CCA
Pro UGG
Ser GGA
Gly GCC
Asp GUC
Leu CAA
Val GAC
Tyr GAU
Asn GUU
Ala GGC
Leu CAG
Leu UAG
Thr GGU
Glu CUC
5s
Phe GAA
Gln UUG
Leu GAG
Leu UAA
Met CAU
Glu UUC

B

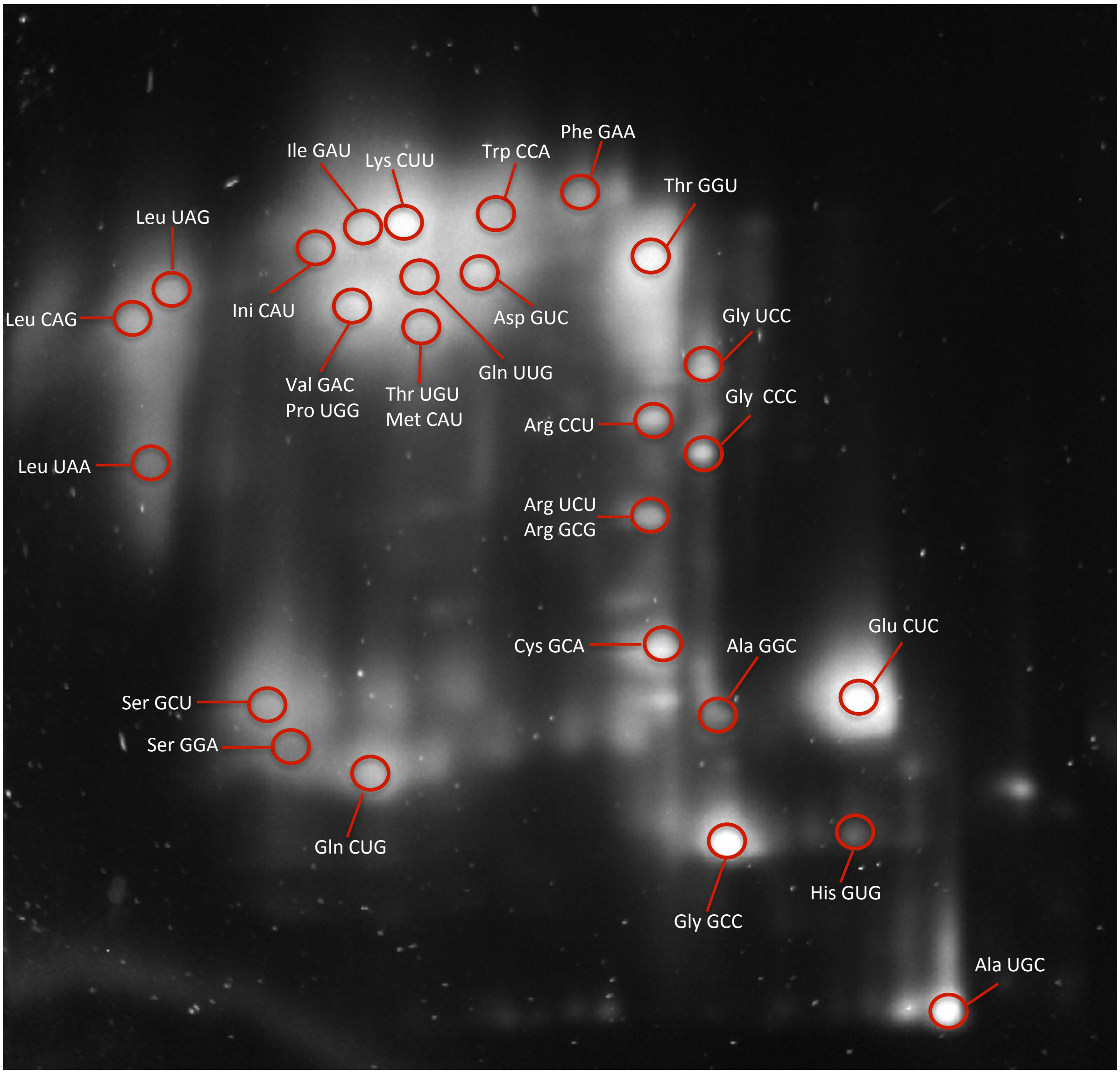

C

Figure S1

20% 2D polyacrylamide gel of total tRNAs. The spots containing tRNAs that were identified by MS/MS sequencing are circled in red. A) *M. maripaludis;* B) *S. acidocaldarius;* C) *P. furiosus.*

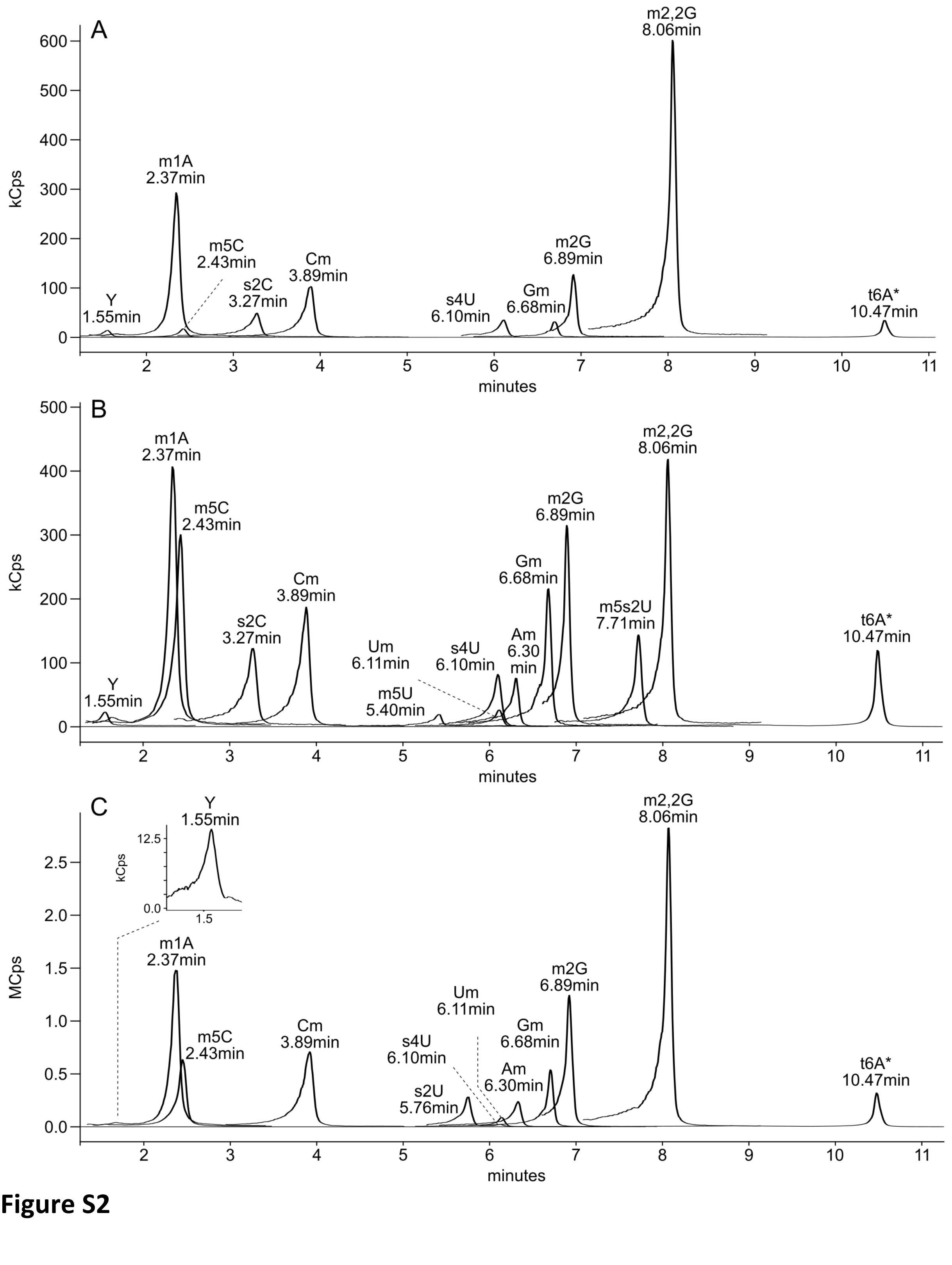

Figure S2

Extracted ions chromatograms for the following 15 nucleosides: m^1A, m^2G, Am, Cm, Gm, Um, s^2C, s^2U, s^4U, m^5s^2U, m^5C, m^5U, $m^2{}_2G$, Y and t^6A. (*) For t^6A, the modifications are analyzed by following only the precursor m/z 413 and the product m/Z 281. A) *M. maripaludis*; B) *P. furiosus*; C) *S. acidocaldarius*. Notice that the relative amount of pseudouridine (Y) in *S. acidocaldarius* is remarkably low compared to *M. maripaludis* and *P. furiosus*. Also, m^5s^2U is absent in the profile of *S. acidocaldarius* compared to *P furiosus.*

a)

	m1A	m2G	Am	Cm	Gm	Um	s2C	s2U	s4U	m5s2U	m5C	m5U	m2,2G	Y
M. maripaludis	1,20E+06	4,97E+05	0,00E+00	2,60E+05	1,28E+05	0,00E+00	1,41E+05	0,00E+00	1,23E+05	0,00E+00	7,98E+04	0,00E+00	2,39E+06	3,26E+04
	1,21E+06	4,83E+05	0,00E+00	2,85E+05	1,27E+05	0,00E+00	1,39E+05	0,00E+00	1,38E+05	0,00E+00	8,44E+04	0,00E+00	2,53E+06	3,28E+04
	1,53E+06	5,65E+05	0,00E+00	4,63E+05	1,43E+05	0,00E+00	2,13E+05	0,00E+00	1,76E+05	0,00E+00	8,70E+04	0,00E+00	2,87E+06	3,21E+04
standard deviation	1,85E+05	4,37E+04	0,00E+00	1,10E+05	8,64E+03	0,00E+00	4,19E+04	0,00E+00	2,75E+04	0,00E+00	3,61E+03	0,00E+00	2,47E+05	3,83E+02
P. furiosus	2,07E+06	1,35E+06	3,89E+05	6,48E+05	9,33E+05	1,29E+05	5,00E+05	0,00E+00	3,58E+05	6,64E+05	1,69E+06	7,52E+04	1,91E+06	6,01E+04
	1,82E+06	1,23E+06	3,63E+05	5,76E+05	8,44E+05	1,18E+05	3,70E+05	0,00E+00	3,28E+05	5,76E+05	1,52E+06	6,92E+04	1,85E+06	5,66E+04
	2,06E+06	1,19E+06	3,54E+05	5,78E+05	8,23E+05	1,26E+05	4,17E+05	0,00E+00	3,21E+05	6,11E+05	1,62E+06	6,63E+04	1,89E+06	5,88E+04
standard deviation	1,44E+05	8,17E+04	1,81E+04	4,10E+04	5,85E+04	5,63E+03	6,54E+04	0,00E+00	1,95E+04	4,40E+04	8,84E+04	4,55E+03	3,12E+04	1,76E+03
S. acidocaldarius	5,65E+06	4,90E+06	1,05E+06	1,79E+06	1,79E+06	3,36E+05	0,00E+00	9,59E+05	3,18E+04	0,00E+00	2,77E+06	0,00E+00	1,12E+07	3,98E+04
	5,58E+06	4,86E+06	1,06E+06	1,97E+06	1,90E+06	3,70E+05	0,00E+00	1,01E+06	3,11E+04	0,00E+00	2,83E+06	0,00E+00	1,09E+07	3,88E+04
	6,89E+06	6,08E+06	1,07E+06	1,92E+06	1,77E+06	3,40E+05	0,00E+00	1,04E+06	3,23E+04	0,00E+00	2,99E+06	0,00E+00	1,12E+07	3,79E+04
standard deviation	7,39E+05	6,94E+05	9,61E+03	9,43E+04	7,23E+04	1,87E+04	0,00E+00	3,84E+04	5,97E+02	0,00E+00	1,15E+05	0,00E+00	1,57E+05	9,34E+02

b)

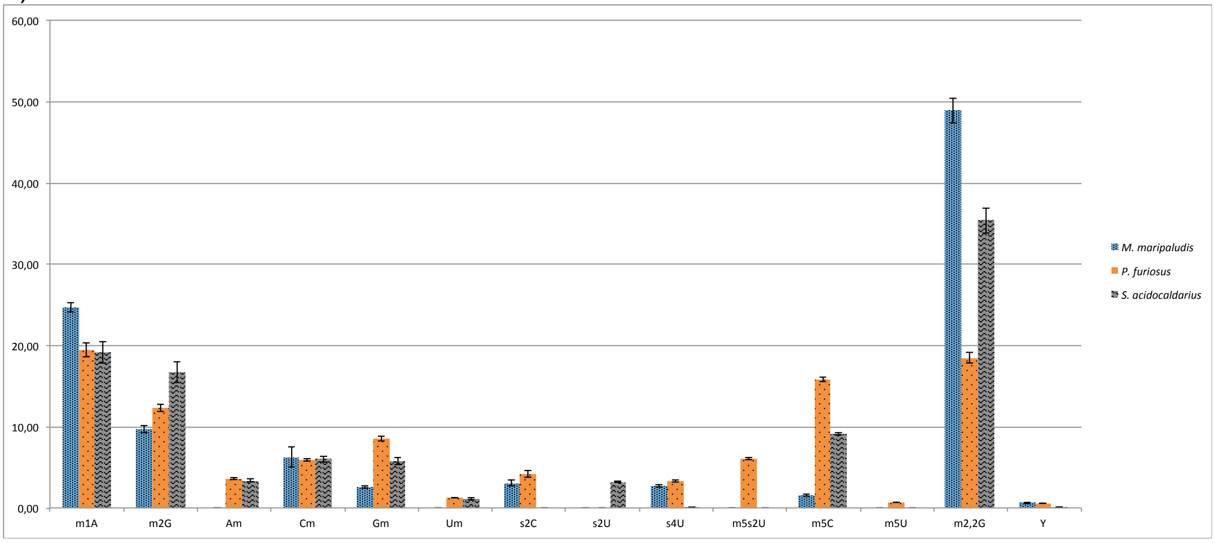

Figure S3

Relative quantification of nucleosides by MRM (multiple reaction monitoring). a) Table of area of nucleosides for each species. The analysis was performed with three technical replicates. b) Relative quantification histogram of nucleoside MRM analysis. Values are normalized by using the sum of area per nucleoside for each archaeon (light blue *M. maripaludis*, orange for *P. furiosus*, and grey for *S. acidocaldarius*).

A

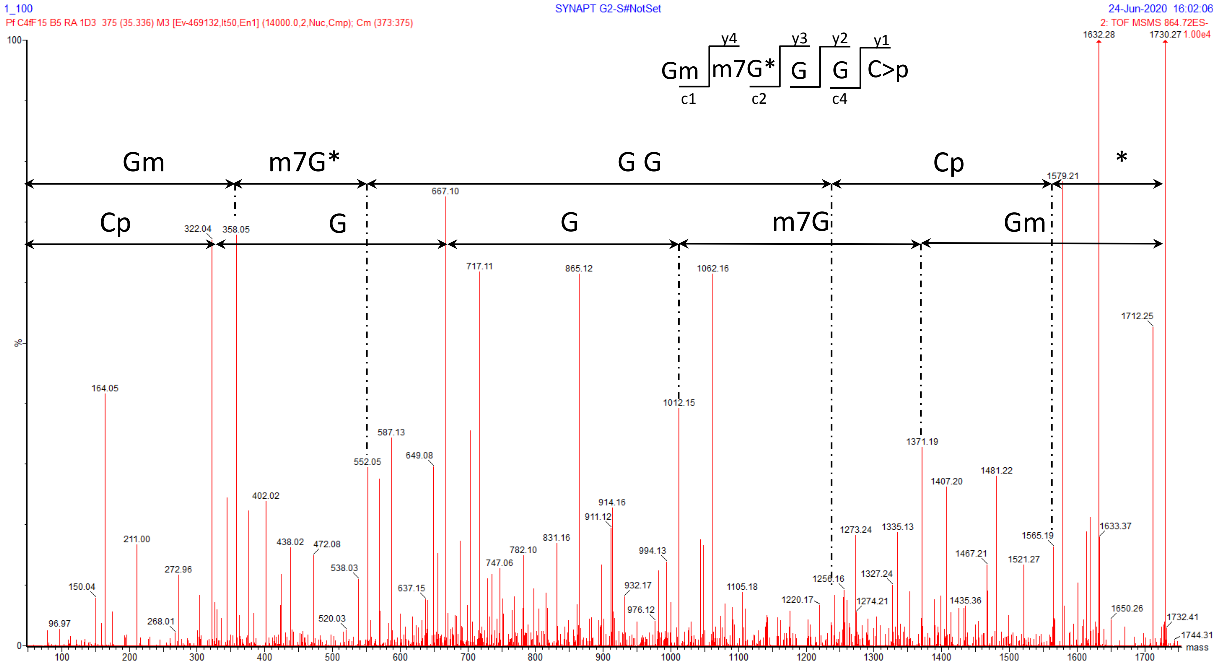

1_100
SYNAPT G2-S#NotSet
24-Jun-2020 16:02:06
2: TOF MSMS 864.72ES-
Gm
m7G*
G
G
C>p
y4
y3
y2
y1
c1
c2
c4
Gm
m7G*
G G
Cp
*
Cp
G
G
m7G
Gm
mass

B

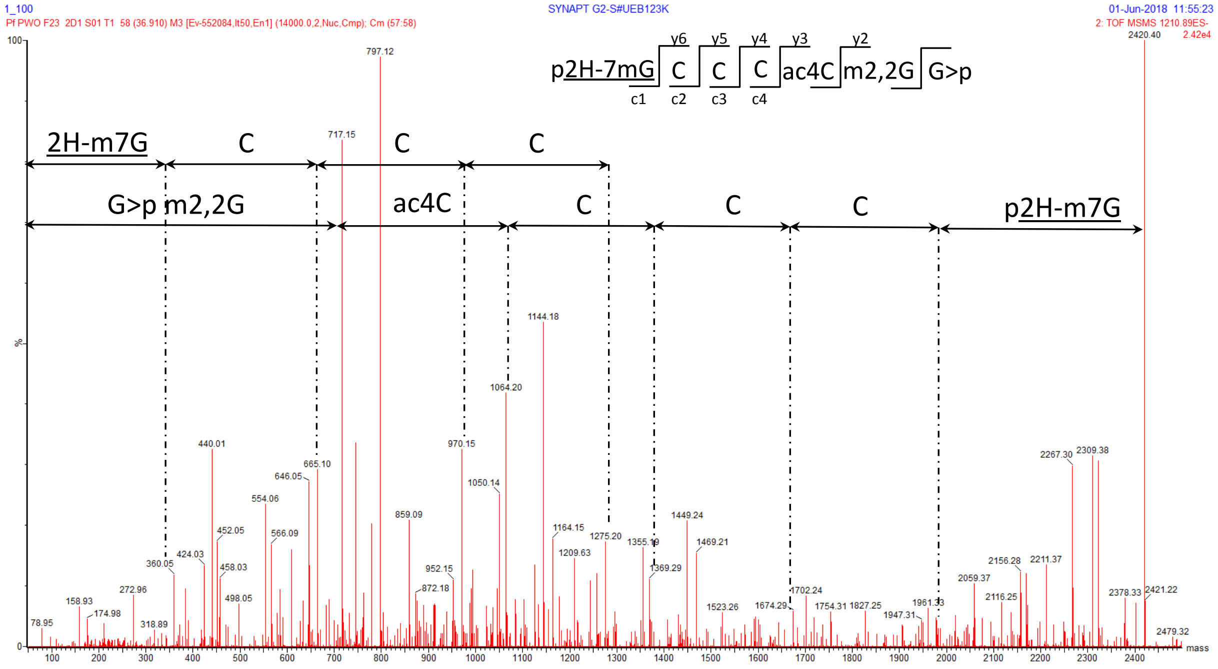

1_100
SYNAPT G2-S#UEB123K
01-Jun-2018 11:55:23
2: TOF MSMS 1210.89ES-
p2H-7mG
C
C
C
ac4C
m2,2G
G>p
y6
y5
y4
y3
y2
c1
c2
c3
c4
2H-m7G
C
C
C
G>p
m2,2G
ac4C
C
C
C
p2H-m7G
mass

C

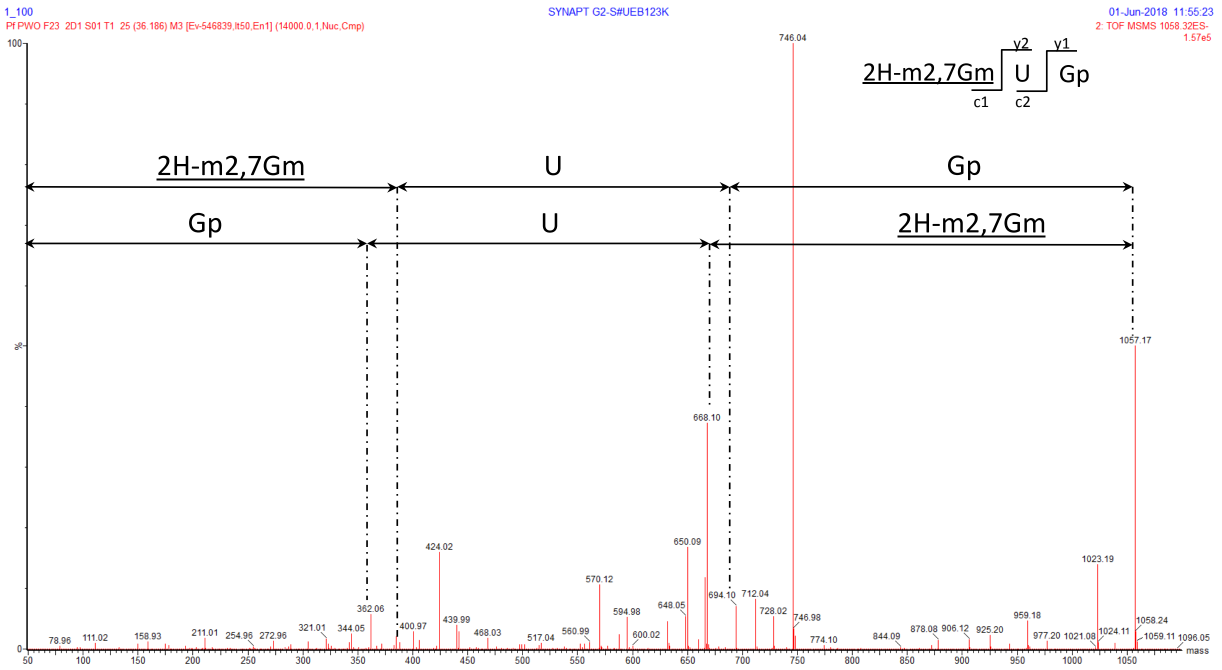

D

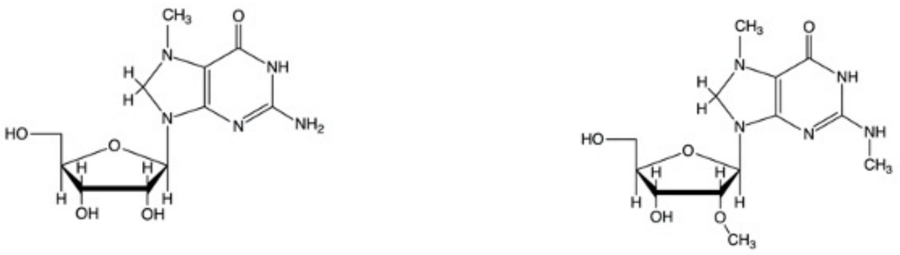

Figure S4

MS/MS sequencing spectra containing m^{7}G. A) MS/MS spectrum [Gm][m7G]GGC>p of tRNA^{ini} of *P. furiosus* after RNase A digestion (m/z 864.63, z = 2-). The spectrum shows a neutral loss of 165 Da, which is specific to m^{7}G and corresponding to the loss of the positively charged modified base. The mass of m^{7}G nucleotide is 359 Da. B) MS/MS spectrum p[2H-m7G]CCC[ac4C]G>p of tRNA^{Glu} of *P. furiosus* after RNase T1 digestion (m/z 1209.66, z = 2-). The mass of m^{7}G nucleotide is 361 Da which could correspond to the reduced form of m^{7}G (Wintermeyer and Zachau 1975). C) MS/MS spectrum [2H-m$^2{}_7$Gm]UGp of tRNA^{Glu} of *P. furiosus* after RNase T1 digestion (m/z 1057.17, z = 1-). The mass of 2H-m$^2{}_7$Gm nucleotide is

389 Da which could correspond to the reduced form of $m^2{}_7Gm$. 2H-m7G and 2H-m2,7Gm are underlined because they cannot be unambiguously characterized by the techniques used and unknown modified guanosine nucleotides cannot be excluded. D) The chemical structures of the reduced forms of m7G derivatives.

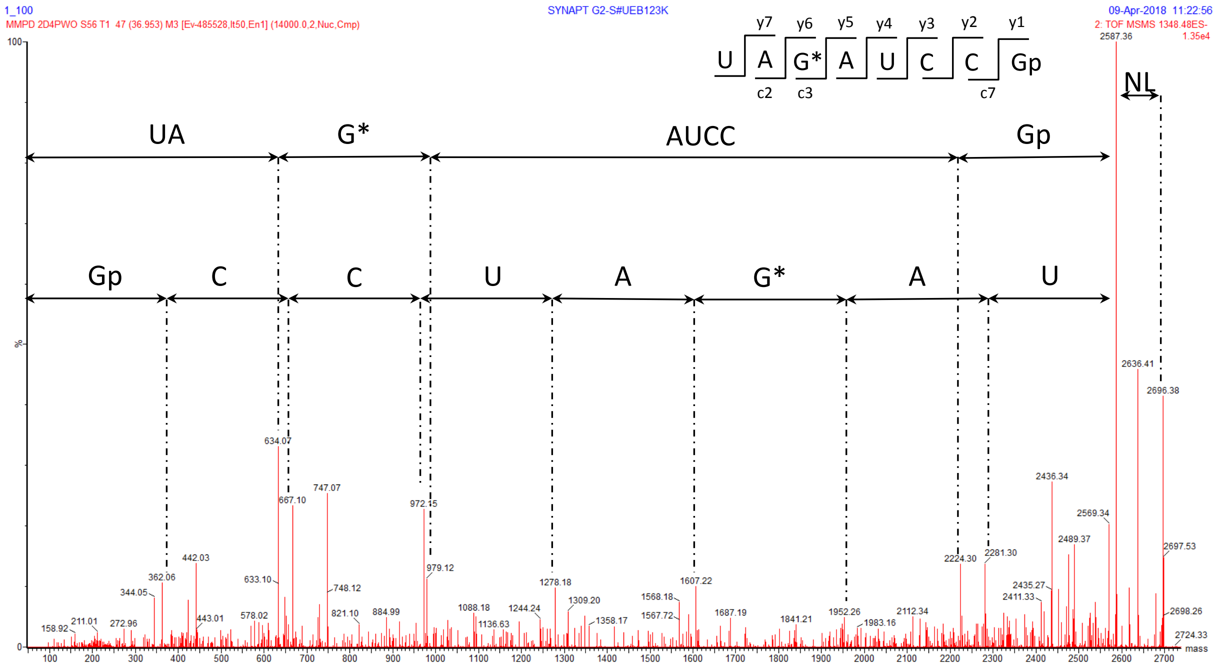

Figure S5

MS/MS sequencing spectrum UA[xG]AUCCGp of $tRNA^{Tyr}$ of *M. maripaludis* after RNase T1 digestion (m/z 1347.71, z = 2-). The spectrum shows a neutral loss of 109 Da corresponding to the loss of an unknown modification from G37. The mass of xG nucleotide is 454 Da. * corresponds to the probable location of the neutral loss.

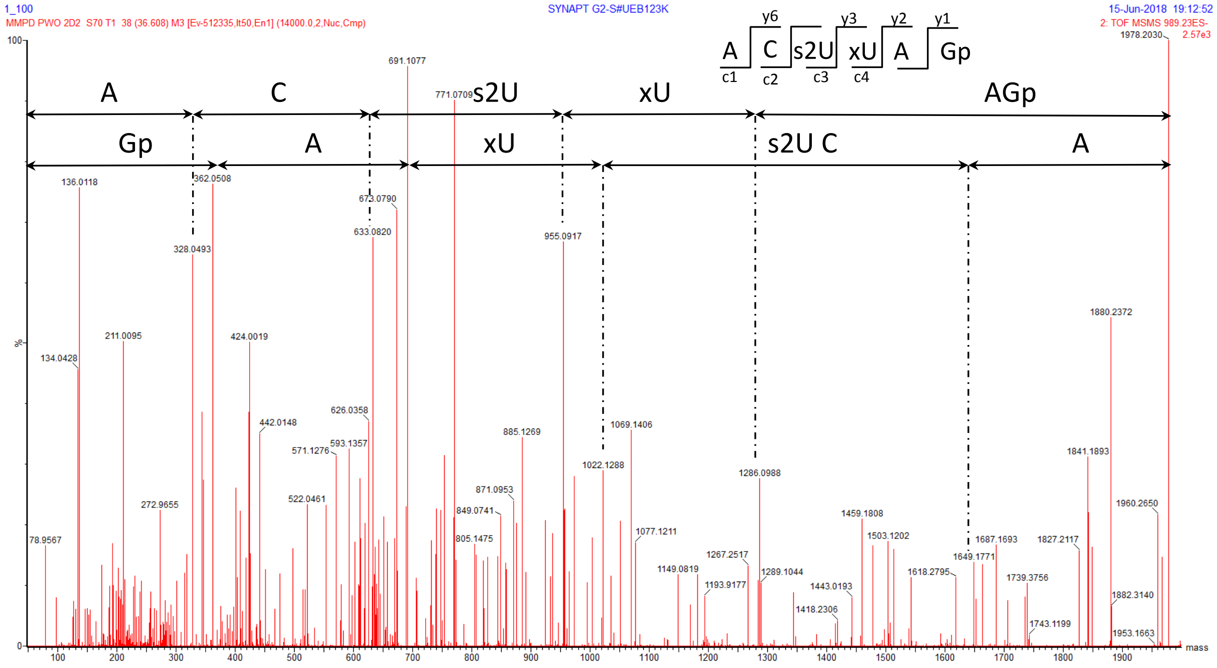

Figure S6

MS/MS sequencing spectrum AC[s^{2}U][xU]AGp of tRNA^{Leu} of *M. maripaludis* after RNase T1 digestion (m/z 988.61, z = 2-). The mass of xU nucleotide is 331 Da.

A

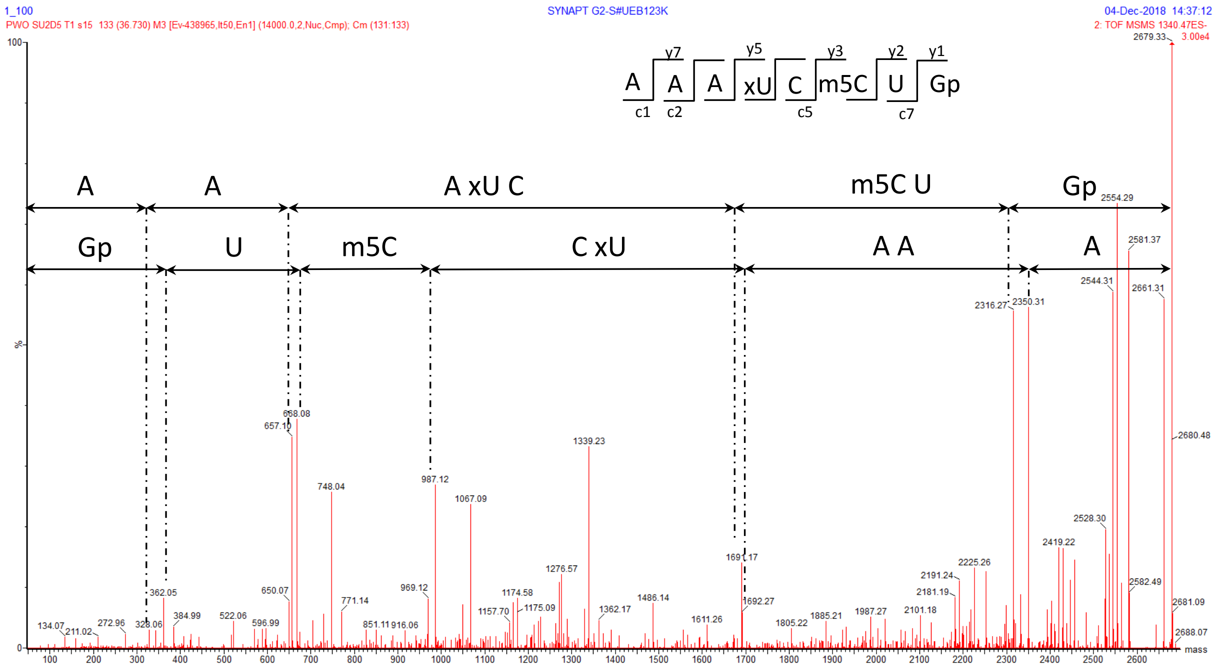

1_100
PWO SU2D5 T1 s15 133 (36.730) M3 [Ev-438965,It50,En1] (14000.0,2,Nuc,Cmp); Cm (131:133)
SYNAPT G2-S#UEB123K
04-Dec-2018 14:37:12
2: TOF MSMS 1340.47ES-
3.00e4
A A A xU C m5C U Gp
y7 y5 y3 y2 y1
c1 c2 c5 c7
A
A
A xU C
m5C U
Gp
Gp
U
m5C
C xU
A A
A
2679.33
2554.29
2581.37
2544.31
2661.31
2316.27
2350.31
2680.48
658.08
657.10
1339.23
748.04
987.12
1067.09
2528.30
2419.22
1691.17
2225.26
1276.57
2191.24
362.05
650.07
969.12
2181.19
2582.49
1174.58
1486.14
771.14
1692.27
2681.09
1157.70
1175.09
1362.17
2101.18
1987.27
1885.21
134.07
272.96
384.99
522.06
1611.26
1805.22
211.02
328.06
596.99
851.11
916.06
2688.07
100 200 300 400 500 600 700 800 900 1000 1100 1200 1300 1400 1500 1600 1700 1800 1900 2000 2100 2200 2300 2400 2500 2600

B

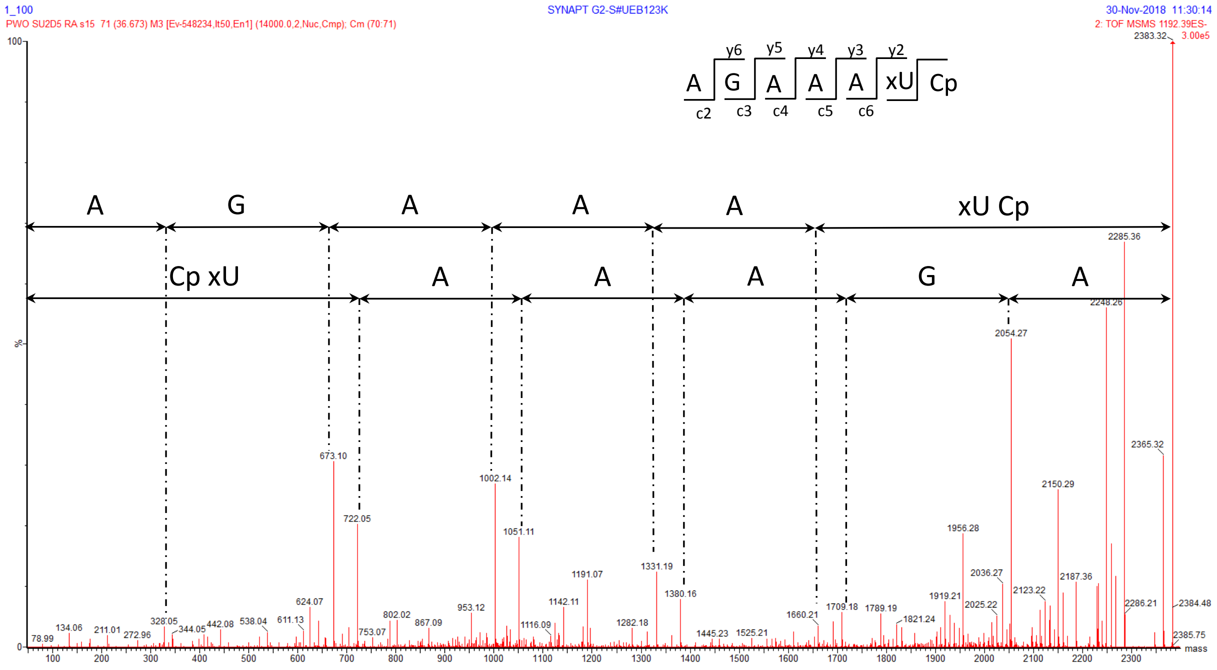

1_100
PWO SU2D5 RA s15 71 (36.673) M3 [Ev-548234,It50,En1] (14000.0,2,Nuc,Cmp); Cm (70:71)
SYNAPT G2-S#UEB123K
30-Nov-2018 11:30:14
2: TOF MSMS 1192.39ES-
3.00e5
A G A A A xU Cp
y6 y5 y4 y3 y2
c2 c3 c4 c5 c6
A
G
A
A
A
xU Cp
Cp xU
A
A
A
G
A
2383.32
2285.36
2248.26
2054.27
2365.32
673.10
1002.14
2150.29
722.05
1956.28
1051.11
1331.19
1191.07
2036.27
2187.36
1380.16
2123.22
1142.11
1919.21
624.07
953.12
2025.22
2286.21
2384.48
1709.18
1789.19
802.02
1660.21
328.05
538.04
611.13
1282.18
1821.24
134.06
442.08
867.09
1116.09
344.05
211.01
753.07
1445.23
1525.21
2385.75
272.96
78.99
100 200 300 400 500 600 700 800 900 1000 1100 1200 1300 1400 1500 1600 1700 1800 1900 2000 2100 2200 2300

C

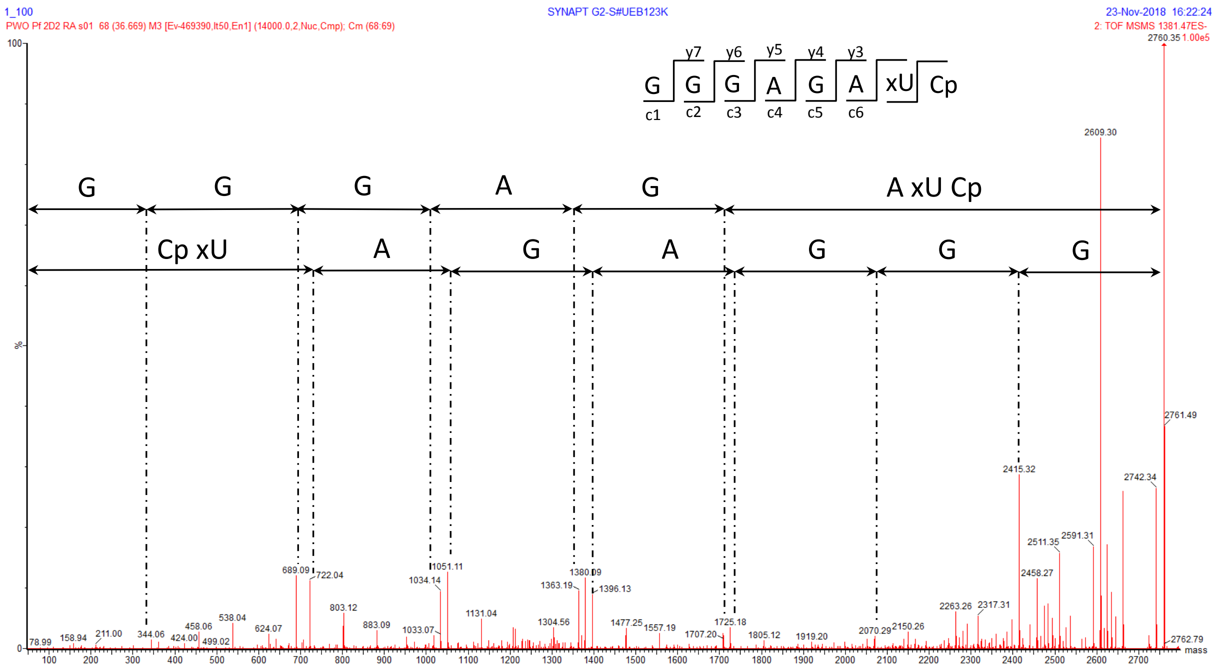

Figure S7

MS/MS sequencing spectra containing xU47 (nucleotide mass of 400 Da). A) MS/MS sequencing spectrum AAA[xU]C[m5C]UGp of tRNA^{Val} of *S. acidocaldarius* after RNase T1 digestion (m/z 1339.17, z = 2-). B) MS/MS sequencing spectrum AGAAA[xU]Cp of tRNA^{Val} of *S. acidocaldarius* after RNase A digestion (m/z 1191.14, z = 2-). C) MS/MS sequencing spectrum GGGAGA[xU]Cp of tRNA^{Met} of *P. furiosus* after RNase A digestion (m/z 1379.67, z = 2-).

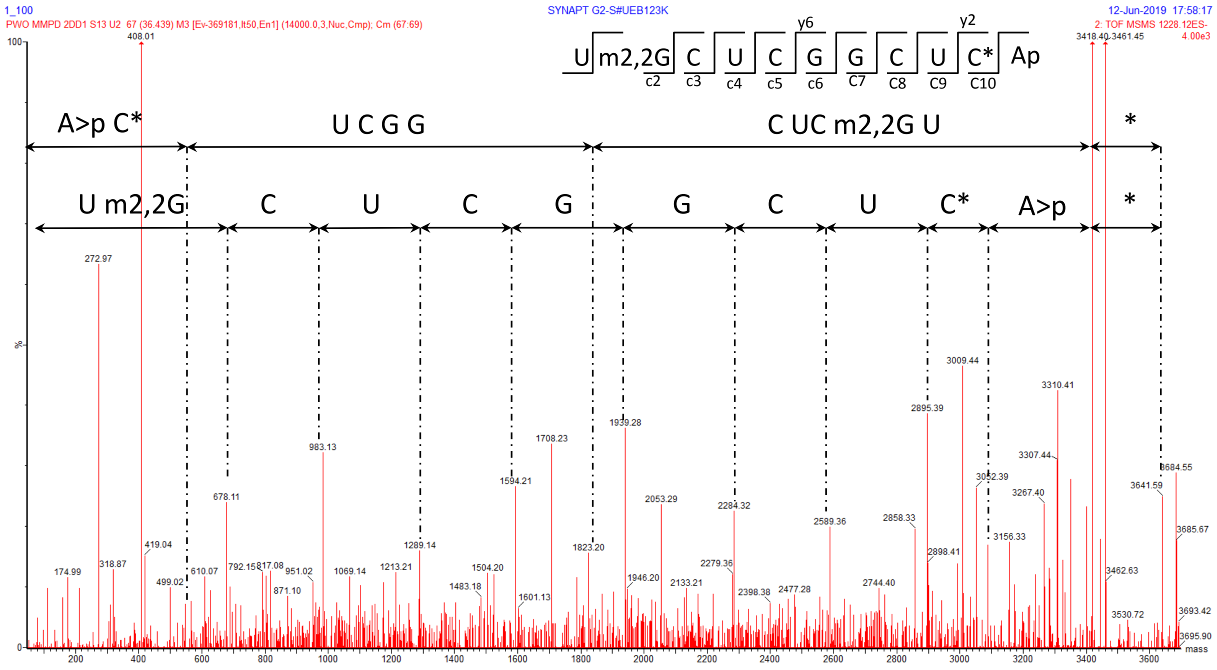

Figure S8

MS/MS sequencing spectrum U[m$^2_2$Gm]CUCGGCU[C+]A>p of tRNA^{Ile} of *M. maripaludis* after RNase U2 digestion (m/z 1227.51, z = 3-). The spectrum shows a neutral loss of 223 Da, which corresponds to the loss of the modification from the cytosine base.

A

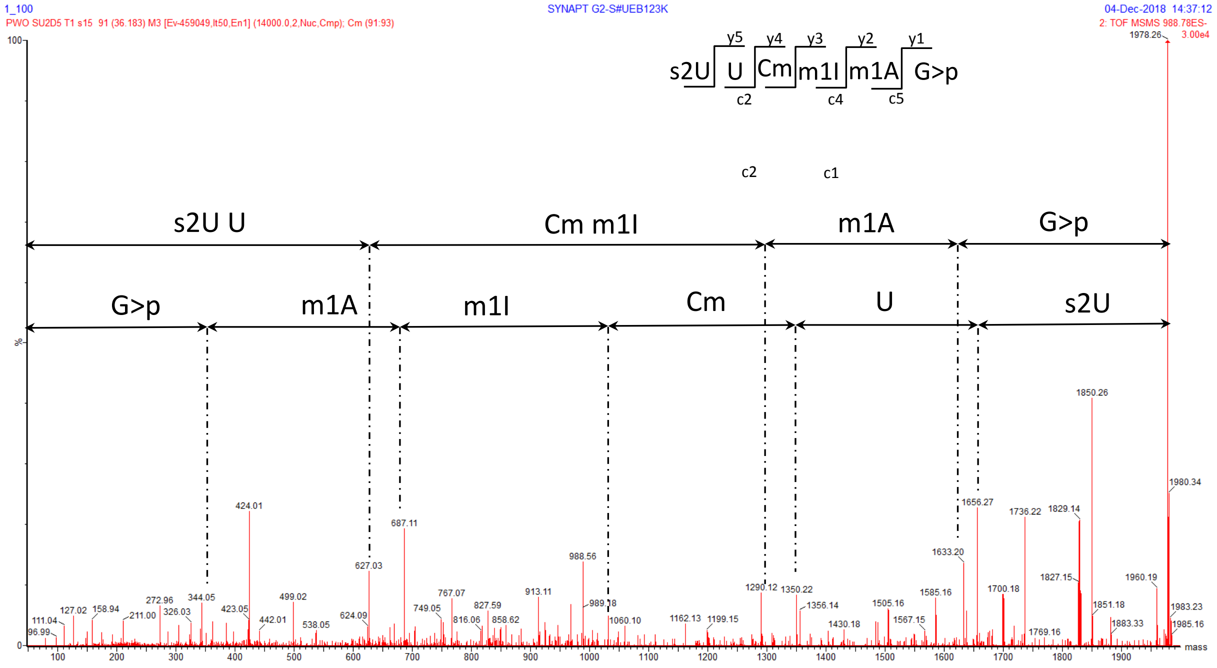
1_100
PWO SU2D5 T1 s15 91 (36.183) M3 [Ev-459049,It50,En1] (14000.0,2,Nuc,Cmp); Cm (91:93)
SYNAPT G2-S#UEB123K
04-Dec-2018 14:37:12
2: TOF MSMS 988.78ES-
3.00e4
s2U U Cm m1I m1A G>p
y5 y4 y3 y2 y1
c2 c4 c5
c2
c1
s2U U
Cm m1I
m1A
G>p
G>p
m1A
m1I
Cm
U
s2U
mass

B

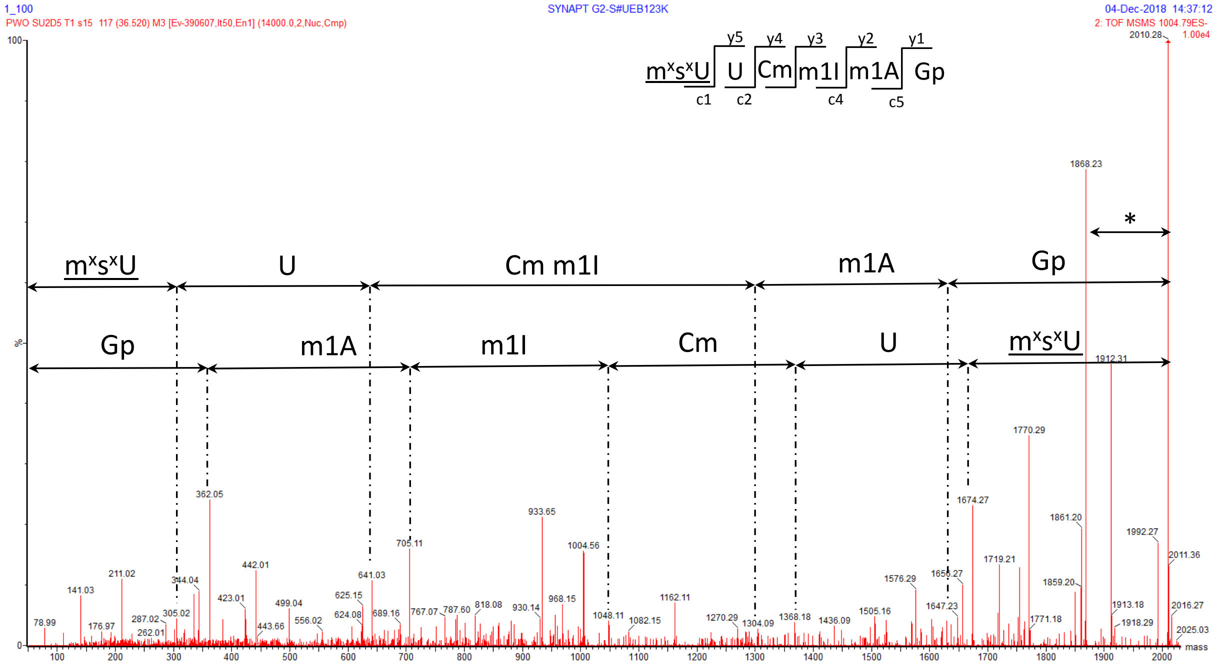
1_100
PWO SU2D5 T1 s15 117 (36.520) M3 [Ev-390607,It50,En1] (14000.0,2,Nuc,Cmp)
SYNAPT G2-S#UEB123K
04-Dec-2018 14:37:12
2: TOF MSMS 1004.79ES-
1.00e4
m^{x}s^{x}U U Cm m1I m1A Gp
y5 y4 y3 y2 y1
c1 c2 c4 c5
*
m^{x}s^{x}U
U
Cm m1I
m1A
Gp
Gp
m1A
m1I
Cm
U
m^{x}s^{x}U
mass

C

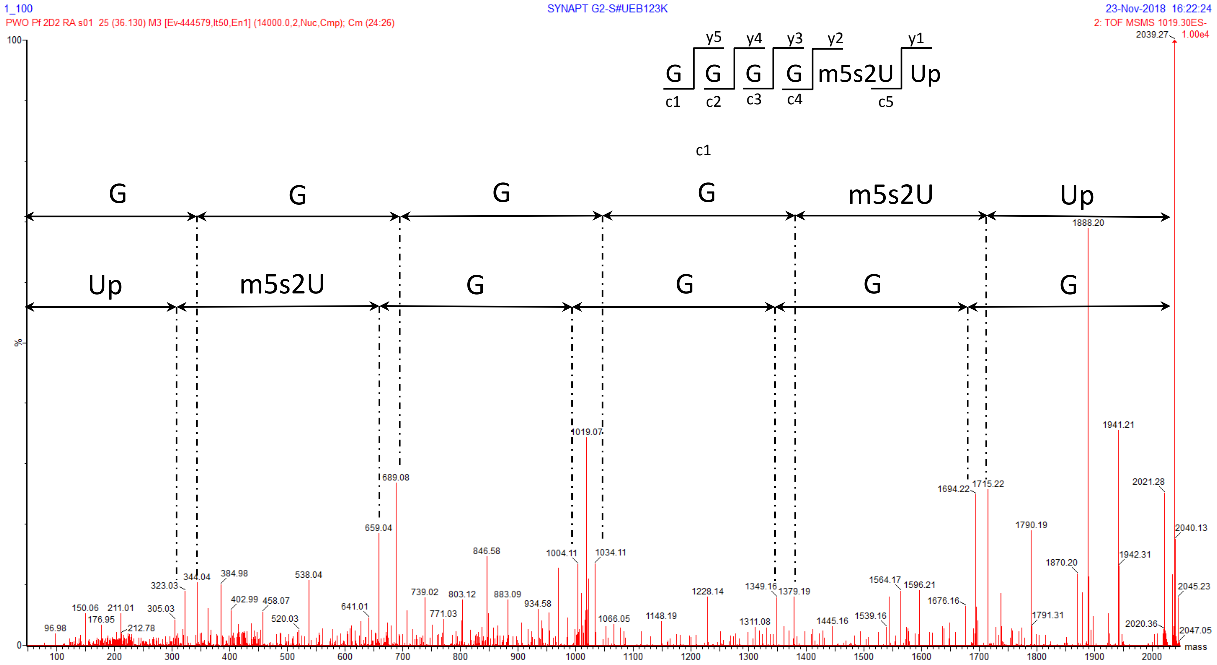

D

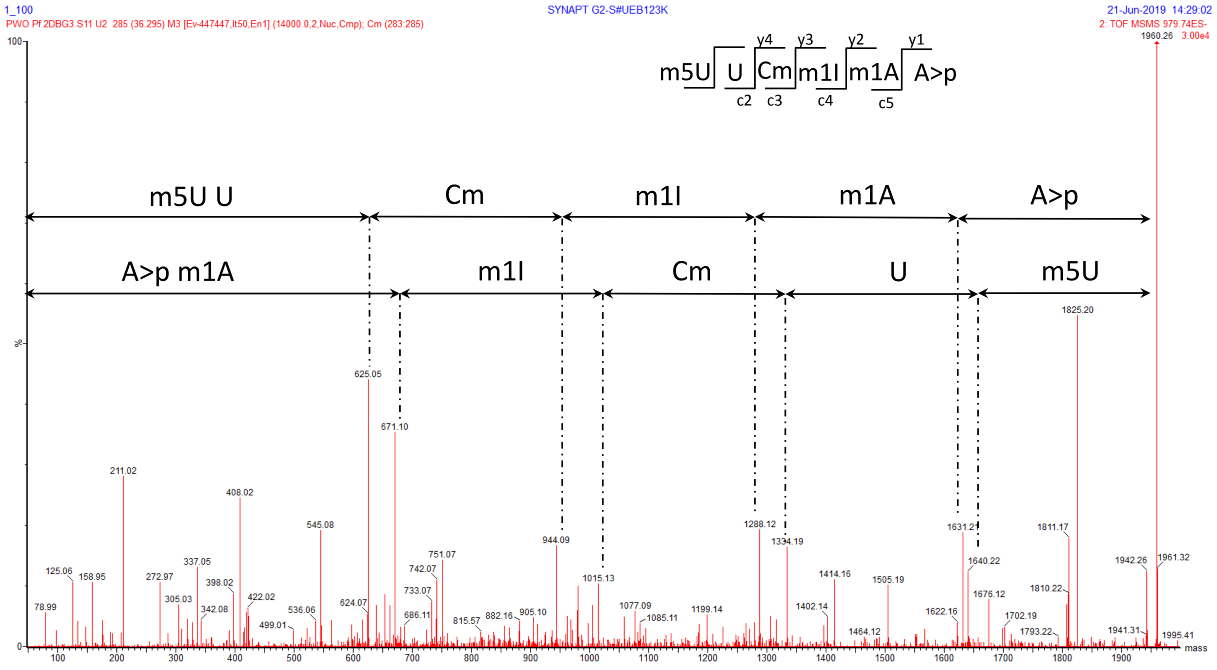

Figure S9

MS/MS sequencing spectra containing U54. A) MS/MS spectrum [s^{2}U]U[Cm][m^{1}I][m^{1}A]G>p of tRNA^{Val} of *S. acidocaldarius* after RNase T1 digestion (m/z 988.62, z = 2-). B) MS/MS spectrum [s^{x}m^{x}U]U[Cm][m^{1}I][m^{1}A]Gp of tRNA^{Val} of *S. acidocaldarius* after RNase T1 digestion (m/z 1004.65, z = 2-). The spectrum shows a neutral loss of 142 Da, which corresponds to the loss of a methyl-thio uracil (or pseudouracil) base. [s^{x}m^{x}U] is underlined because the position

of methyl and thio groups are unambiguously localized. C) MS/MS spectrum GGGG[m^5s^2U]Up of $tRNA^{Met}$ of *P. furiosus* after RNase A digestion (m/z 1019.12, z = 2-). D) MS/MS sequencing spectrum [m^5U]U[Cm][m^1I][m^1A]A>p of $tRNA^{Lys}$ of *P. furiosus* after RNase A digestion (m/z 979.63, z = 2-).

Methanococcus maripaludis

AA AC	Acc-5'		D-5'	D-loop			D-3'		AC-5'	Ac-loop				AC-3'	V-region		T-5'	T-loop				T-3'	Acc-3'	
	1	8	1	1			2	2	2	3				3	4	4	4	5				6	6	7
			0	4			2	6	7	2				9	4	8	9	4				1	6	3
H GUG	GCCGUGG	UA	GGGU	AG	U	GGCU A	UCCU	G	AGGGA	CU	GUG	m1G	A	UCCCU	CGA	C	CCGGG	UUC	G	A	UU	CCCGG	UCUCGGC	C
L UAG	GCAGGGG	UU	GUCG	AG+	CCU	GGCCAA	AGAU	*G	CAGGA	Cs2U	xUAG	A	A	UCCUG	UCCAGUAGUGGUU	C	CAGGG	UUC	A	A	AU	CCCUG	CCCCUGC	A
L GAG	GCGAGGG	UU	GCCA	AG+	Cs2CU	GGUUAA	AGGC	*G	CUAGG	UU	GAG	G	G	CCUAG	UCUCAUAGGAGUU	C	GAGGG	UUC	G	m1A	AU	CCCUU	CCCUCGC	
P UGG	GGGCCUG	UG	GGGU	AG+	CCU	GGUC A	UCCU	U	UGGGA	UU	UGG	m1G	A	UCCUG	AAA	C	CCCAG	UUC	G	m1A	AU	CUGGG	CAGGCCC	A
A UGC	GGGCCCG	UA	GCUC	AG+	UU	GG A	GmAGC	G	CCGCC	CU	UmG[C/*C]	A	A	GGCGG	AGCU	C	GUGGG	m1YUCm	m1I	m1A	AU	CCCGC	CUAGGCU	
G GCC	GCGGCUU	UG	AUGU	AG+	ACU	GGU A	UCAU	A	CGGCC	CU	GCC	A	C	GGCCG	ACA	C	CCGGG	UUC	A	A	AU	CCCGG	AGGCCGC	
G UCC	GCGCCAG	UG	GUGU	AG	CCU	GGU A	UCAC	U	UUGGC	CU	cnm5UCC	A	A	GCCAG	UAA	C	UCGGG	m1YUCm	m1I	m1A	AU	CCCGA	CUGGCGC	A
D GUC	GCUCUGG	UG	GUGU	AG+	CUC	GGCCUA	UCAU	A	CAGGA	CU	GUC	f6A	C	UCCUG	UGA	C	UCGGG	m1YUCm	m1I	m1A	AU	CCCGG	CCAGAGC	G
E UUC	GCUCCAG	UG	*GUGU	AG	UCC	GGCCAA	UCAU	C	CGGCC	CU	mnm5UUC	m1G	A	GGCCG	GGA	C	UCGGG	m1YUCm	m1I	m1A	AU	CCCGG	CUGGAGC	A
V UAC	GGACCU*A	UG	GUCU	AG+	UU	GGCU A	UGAC	A	UCGCC	CU	UAC	A	A	GGCGA	GGGU	C	GCCGG	UUC	G	A	AU	CCGGC	UAGGUCC	A
V GAC	GAGUUCG	UG	GUCU	AG+	UU	GGCU A	UGAU	A	CCGCC	CU	GAC	A	C	GGCGG	UGAU	C	GGGAG	UUC	G	m1A	AU	CUCCC	CGGACUC	A
F GAA	GCCAAGG	UA	GUUC	AG	CCU	GGG A	GAAC	*G	CUGGA	CmU	GAA	imG-14	A	UCCAG	UUGU	C	GGGUG	UUCm	m1I	m1A	AU	CACCC	CCUUGGC	A
L UAA	GGAGGGA	UU	GCCA	AG	CCU	GGUCAA	AGGC	G	UCGGA	CU	UAA	G	A	UCCGA	UCCGGUAGCGGUU	C	GAGGG	UUC	A	A	AU	CCCUU	UCCCUCC	A
S UGA	GCAGAGG	UG	GUUG	AG+	CUU	GGCCAA	AGGC	*G	CCGGA	CU	UGA	A	A	s2UCCGG	UUCUCCACUGGGGAG	C	GGGGG	m1YUCm	m1I	m1A	AU	CCCUC	CCUCUGC	
S GGA	GCAGAGA	UA	GUCU	AG+	CCU	GGG A	AGGC	*G	UACGG	CU	GGA	A	A	CCGUA	UGAGGCUUUGCCUCC	C	GGGGG	UUC	A	A	AU	CCCCC	UCUCUGC	G
Y GUA	CCCGCGA	UA	GUUC	AG+	ACU	GGU A	GAAC	*G	GCGGA	CmU	GUA	xG	A	UCCGC	AUGU	C	GCUGG	m1YUCm	m1I	m1A	AU	CCGGC	UCGCGGG	A
W CCA	GGGAGUA	UG	GUGU	AG+	Us2CU	GGUCUA	UCAU	C	GGGGA	CU	CCA	A	A	UCCCU	GGA	C	CUGGG	UUC	A	A	AU	CCCAG	UACUUCC	A
I GAU	AGGGCAG	UG	GCUC	AG+	Cs2CU	GGUU A	GAGU	*G	CUCGG	CU	GAU	hn6A	A	CCGAG	UGGU	C	CGGGG	UUCm	G	m1A	AU	CCCCG	CUGCCCU	A
I CAU	GGGCCCG	UA	GCUC	AG	GCU	GGUU A	GAGU	*G	CUCGG	CU	C+AU	hn6A	A	CCGAG	UGGU	C	AUGGG	m1YUCm	m1I	m1A	AU	CCCAU	CGGGCCC	A
K UUU	GGGCCCG	UA	GCUU	AG+	UCU	GGU A	GAGC	*G	CCUGA	CU	cmnm5s2UUU	t6A	A	UCAGG	CGGU	C	GAGGG	UUC	G	m1A	AU	CCCUU	CGGGCCC	G
Mi CAU	AGCGAGG	UA	GGGU	AG+	CCA	GGCCUA	UCCC	G	CCGGG	CU	CAU	A	A	CCCGG	AGAU	C	AGAAG	UUC	A	A	AU	CUUCU	CCUCGCU	A
M CAU	GCCGAGG	UG	GCUU	AG+	GCU	GGUU A	UAGC	*G	CUCGG	CU	CmAU	hn6A	A	CCGAG	AGGU	C	GGGGG	m1YUCm	m1I	A	GU	CCCCC	CCUCGGC	
N GUU	GCCUCCU	UA	GCUC	AG+	UA	GGUA G	CAGC	*G	ACGGA	CU	GUU	hn6A	A	UCCGU	AGGU	C	GCAGG	UUC	G	A	GC	CCUGC	AGGAGGC	G
R UCU	GGGCCCG	UG	GCCU	AG	UCU	GGAU A	CGGC	A	CCGGC	CU	UCU	t6A	A	GCCGG	GGAU	C	GGGGG	m1YUC	G	m1A	AU	CCCUC	CGGGUCC	G
S GCU	GCGGAGA	UG	GCCC	AG+	UCU	GGU A	CGGC	G	CGGGA	CU	GCU	t6A	A	UCCCG	UUACGCUCUGCGUAA	C	CCGAG	UUC	A	A	AU	CUCGG	UCUCCGC	
T UGU	GCCUCGG	UG	GCUC	AG	CCU	GGU A	GAGC	G	CCUGA	CU	UGU	hn6A	A	UCAGG	UGGU	C	GGGGG	UUC	G	A	AU	CCCCC	CCGGGGC	U
T GGU	GCUUCAG	UG	GCUC	AG+	CCU	GGU A	GAGC	*G	CCUGC	UU	GGU	hn6A	A	GCAGG	AGGC	C	GCGGG	UUC	A	A	AC	CCCGC	CUGAAGC	U
	1	8	1	1			2	2	2	3				3	4	4	4	5				6	6	7
			0	4			2	6	7	2				9	4	8	9	4				1	6	3
	....	UR	G..Y	AG		GG R	...Y	R	...G.	CU			A	.C...		C	....G	UUC	R	m1A	RY	C....	....	R

Pyrococcus furiosus

AA AC	Acc-5'		D-5'	D-loop			D-3'		AC-5'	Ac-loop				AC-3'	V-region		T-5'	T-loop				T-3'	Acc-3'	
	1	8	1	1			2	2	2	3				3	4	4	4	5				6	6	7
			0	4			2	6	7	2				9	4	8	9	4				1	6	3
L UAG	GCGGGGG	UU	GCCG	AG+	CCU	GGUCAA	AGGC	*Gm	CGGGA	UU	cnm5UAG	G	G	UCCCG	UCCCGUAGGGGUU	C	CGGGG	UUC	A	A	AU	CCCCG	CCCCCGC	A
L CAG	GCGGGGG	UU	GCCG	AG+	Cs2CU	GGUCAA	AGGC	*Gm	CGGGA	UU	CAG	G	G	UCCCG	UCCCGUAGGGGUU	C	CGGGG	UUC	A	A	AU	CCCCG	CCCCCGC	A
P UGG	GGGGCCG	UA	GGGU	AG	CUU	GGCCCA	UCCU	m2G	CGGGC	UU	cnm5UGG	m1G	A	GCC*CG	UGA	C	CCGGG	UUC	A	A	AU	CCCGG	CGGCCCC	A
Q UUG	AGCCCCG	UG	GUGU	AG+	C	GmGCCAA	GCAU	G	CGGGA	CU	cnm5UUG	G	A	UCCCG	CGA	m5C	m5CCGGG	UUC	G	m1A	AU	CC*CGG	CGGGGCU	A
Q CUG	AGCCCCG	UG	GUGU	AG+	C	GmGCCAA	GCAU	G	CGGGA	CU	ac4CmUG	G	A	UCCCG	CGA	m5C	m5CGmGGG	UUC	G	A	AU	CCCCG	CGGGGCU	A
R GCG	GCCCCGG	UG	GCCU	AG	CCU	GGAU A	GGGC	m2G	CGAGG	CU	GCG	mimG	A	CCUCmG	AGGU	C	CGGGG	UUC	A	A	AU	CCCCG	CCGGGCG	C
A UGC	GGGC*Cm2GmG	UA	GCUC	AG+	CCU	GGU AU	GAGC	Gm	CCGCC	CU	cnm5UGC	A	A	GGCGG	AGGC	C	CCGGG	m5UUCm	m1I	m1A	AU	CC*CGG	CCGGUC	A
A GGC	GGGCCGG	UA	GCUC	AG	CCU	GGU AU	GAGC	G	CCGCC	UU	GGC	A	A	GGCGG	AGG	C	CCGGG	UUC	A	A	AU	CCCGG	CCGGUCC	A
D GUC	GCCCGGG	UG	GUGU	AG	CCC	GGCCUA	UCAU	m2G	CGGGA	CU	GUC	A	C	UCCCG	CGA	C	CCGGG	UUC	A	A	AU	CCCGG	CCCGGGC	G
E CUC	m7GCCC*C*GG	UG	m2,7GmUGU	AG	CCC	GGUCAA	UCAU	m2G	CGGGA	CU	CUC	m1G	A	UCCCG	CGA	m5C	m5CCGGG	UUC	A	A	AU	CCCGG	CCGGGGC	A
G CCC	GCGGUGG	UA	GUCU	AG+	Cs2CU	GGUCUA	GGAC	G	CCGGC	CU	CCC	A	A	GCCGG	CGA	m5C	m5CCGGG	UUC	A	A	AU	CCCGG	CCACCGC	A
G UCC	GCGGUGG	UA	GUCU	AG+	CCU	GGUCUA	GGAC	m2G	CCGGC	CU	cnm5UCC	m1G	A	GCCGG	CGA	C	CCGGG	UUC	A	A	AU	CCCGG	CCACCGC	A
G GCC	GCGGUGG	UA	GUCU	AG	CCU	GGUCUA	GGAC	G	CCACC	CU	GCC	A	A	GGUGG	AGA	C	CCGGG	UUC	A	A	AU	CCCGG	CCACCGC	A
V UAC	GGGCCCG	UG	GUCU	AG+	CU	GGUU A	UGAC	G	CCGCC	CU	UAC	G	A	GGCGG	AGGU	C	CGGGG	UUC	G	m1A	AU	CCC*CG	CGGGCCC	A
V GAC	GGGCCCG	UG	GUCU	AG+	ACU	GGUU A	UGAC	*G	CCACC	CU	GAC	A	A	GGUGG	AGGU	C	CGGGG	UUC	A	A	AU	CCCCG	CGGGCCC	A
C GCA	GCCGGm2GmA	Um1A	GCCU	AG+	A	GGC C	AGGC	*Gm	GGGGA	CU	GCA	G	A	UCCCC	UUUA	C	CCGGG	m5s2UUCm	m1I	m1A	AU	CCCGG	UCCCGGC	U
F GAA	GGGGCGG	UA	GCUC	AG+	Cs2CU	GGG A	GAGC	A	CCGGA	CU	GAA	mimG[A/Am]		UCCGG	GUGU	C	GGGGG	UUC	A	A	AU	CCCCC	CCGCCCC	A
L UAA	GCGGGGG	UG	CCCG	AG	CCU	GGCCAA	AGGG	G	CCGGA	CU	UAA	G	A	UCCGG	UGCCGUAGGGCUG	C	GCGGG	UUC	G	A	AU	CCCGC	CCCCCGC	A
S UGA	GCCGGm2G/*GA	U/Umm1A	GCCU	AGCCU		GGU A	GGGC	*Gm	CGGGC	CU	UGA	imG2/mimG	A	GCCCG	UmGGGCGUUUGCCCG	C	CGGGG	m5U/m5s2UUC	A	A	AU	CCCCG	UCCCGGC	G
S GGA	GCCGGGG	UA	GCCU	AG	CCA	GGG A	AGGC	G	CGGGC	CU	GGA	mimG	A	[G/m2,2G]CCCG	UGGGCGTTCGCCCA	C	CGGGG	UUC	A	A	AU	CCCCG	CCCCGGC	G
W CCA	GGGGGCG	UG	GUGU	AG	CCU	GGUCCA	UCAU	C	GCGGG	CU	CCA	mimG	A	CmCCmGmC	GGA	m5C	CGGGG	UUCm	m1I	m1A	AU	CCCCG	CGCCCCC	A
I GAU	GGGCCCG	UG	GCUC	AG+	Cs2CU	GGUC A	GAGC	Gm	CCCGC	CU	GAU	hn6A	A	GCGGG	AGGU	C	CGGGG	UUC	G	A	AG	CCC*CG	CGGGCCC	A
K CUU	GGGCCGG	UA	GCUU	AG	CCU	GGUU A	GAGC	*Gm	GCGGA	CU	CUU	ms2t6A	A	UCCGC	AGGU	C	GGGGG	m5UUCm	m1I	m1A	AU	CCCCC	CCGGCCC	G
Mi CAU	AGCGGm2GG	UGm	m7GGGC/CmAG+		CUA	GGAG	UmGmCCC	*G	CCGGG	CU	CAU	A	A	CCCGG	AGGxU	C	CGAGG	m5s2UUCm	m1I	m1A	AU	CCUCG	GCCCGCU	A
M CAU	GCCGGm2GmG	UA	GCUU	AG	CCU	GGUC A	AAGC	G	CmCCGG	CU	CmAU	hn6A	A	CCGGG	AGAxU	C	CGGGG	m5s2UUCm	G	m1A	AG	CCC*CG	CCCCGGC	A
R CCU	GGAC*Cm2GmG	UA	GCCU	AG+	CCA	GGAU A	GGGC	*Gm	GCGGC	s2CU	CCU	t6A	A	GCCGC	AGGU	C	CGGGG	UUC	A	A	AU	CCCCG	CCGGUCC	G
R UCU	GGGCCCG	UA	GCCU	AG+	CA	GGAU A	GGGC	G	CGGGC	CU	cnm5UCU	A	A	GCCGG	AGGU	C	CCGGG	UUC	G	m1A	AU	CC*CGG	CGGGCCC	G
S GCU	GCCGCGG	UA	GCCU	AG+	CCU	GGU A	GGGC	*Gm	CCGGC	CU	GCU	t6A	Am	GCCGG	UGGGCGUGGUCCCG	C	CGGGG	UUC	A	A	AU	CCCCG	CCGCGGC	G
T UGU	GCCCGGG	UA	GCCU	AG	CCU	GGU G	GGGC	*Gm	GGGGA	CU	cnm5UGU	hn6A	A	UCCCC	AGGU	C	GCGGG	UUC	A	A	AU	CCCGC	CCCGGGC	U
T GGU	GCCCCGG	UG	GCUC	AG	CCU	GGU A	GAGC	*Gm	GCCGC	UU	GGU	[t6A/hn6A]	A	GCGGC	AGGxU	C	CCGGG	UUCm	m1I	m1A	AG	CCCGG	CC[G/Gm]GGGC	U
	1	8	1	1			2	2	2	3				3	4	4	4	5				6	6	7
			0	4			2	6	7	2				9	4	8	9	4				1	6	3
	....	UR	G..Y	AG		GG R	...Y	R	...G.	CU		R	R	.C...		C	....G	UUC	R	m1A	AY	C....	....	R

Sulfolobus acidocaldarius

AA AC	Acc-5'		D-5'	D-loop			D-3'		AC-5'	Ac-loop				AC-3'	V-region		T-5'	T-loop				T-3'	Acc-3'	
	1	8	1				2	2	2	3				3	4	4		5				6	6	7
			4				2	6	7	2				9	4	8		4				1	6	3
L GAG	GCGGGGG	UG	CCCG	AG+	CAmA	GGUCA A	AGGG	*G	GCGGA	CmU	GAG	m1G	C	UCCGC	UGGUGUAGGCCUG	C	GUmGGG	s2UCm	m1I	m1A	GU	CCCAC	CCCCCGC	A
L UAG	GCGGGGG	UG	CCCG	AG	CAA	GGUCA A	AGGG	G	UCGGG	CmU	s2UmAG	m1G	C	CC*CGA	UGGUGUAGGCCUG	C	GUGGG	UUC	A	A	AU	CCCAC	CCCCCGC	A
L CAG	GCGGGGG	UG	CCCG	AG	CAA	GGUCA A	AGGG	G	UCGGG	CmU	CmAG	m1G	C	CCCGA	UGGUGUAGGCCUG	C	GUGGG	UUC	A	A	AU	CCCAC	CCCCCGC	A
P UGG	GGGGCCG	UA	GUCU	AG	CUU	GGAmCU A	GGAU	*G	CCAGG	CU	UGG	m1G	C	CCUGG	UGGU	C	CCGGG	UUC	A	A	AU	CCCGG	CGGCCCC	A
Q UUG	AGCCGGG	UC	GUCU	AG+	C	GmGUCA A	GGmAU	Cm	CAGGG	CU	[Um/s2Um]UG	m1G	C	CCCUG	GGA	m5C	m5CAGGG	UUC	G	A	GU	CCCUmG	CCCGGCUm	A
A GGC	GGGCCGG	UA	GCUC	AG+	CCmU	GGA A	GAGU	*G	CGUGG	UU	GGC	A	U	CCACG	AGGU	C	CCGGG	UUC	A	A	AU	CCCGG	CCGGUCC	A
A UGC	GGGCCGG	UA	GCUC	AG+	CCmU	GGA A	GAGU	*G	CUUGG	CmU	UmGC	A	A	C*CGAG	AGGU	C	CCGGG	UUC	A	A	AU	CCCGG	CCGGUCC	A
G GCC	GCGGCCG	UA	GUCU	AG+	CCU	GGAmUU A	GGAC	*G	CCUGC	CU	GCC	A	C	GCAGG	AGGxU	C	CCmGGG	s2UUCm	m1I	m1A	AU	CCCGG	CGGUCGC	A
V GAC	GGGCCCG	UC	m2GUCU	AG+	CCmU	GGUU A	GGACm	*G	CUGCC	CmU	GAC	m1G	C	GGCAG	AAAxU	C	m5CUmGGG	[sxmxU/Y]UCm	m1I	m1A	GU	CCCAG	CGGGCCC	A
D GUC	GC*CGCGG	Um1A	*GUCU	AG+	C	GGUCU A	GGAUm	*G	GGGGC	CU	GUC	mG/imG2/mimG	A	GCCCU	UGA	m5C	m5CCmGmGG	s2UUCm	m1I	m1A	AU	CCCGG	CCGCmGGm5C	G
E UUC	GCCGCGG	UA	*GUAU	AG+	CCC	GmGUCA A	GUAU	*G	CmGGGC	CU	UUC	G	A	GCCCG	UGA	m5C	m5CCmGGG	UUC	A	A	AU	CCCGG	CCGCGGC	A
E CUC	GCCGCGG	UA	GUAU	AG+	CCC	GmGUC A	GUAU	*G	CmGGGC	CmU	CUC	A	A	GCCCG	UGA	m5C	m5CCGmGG	s2UUCm	m1I	m1A	AU	CCCGG	CCGCGGC	A
F GAA	GCCGCCG	UA	GCUC	AG	CCC	GGG A	GAGCm	*G	CCCGG	CU	GAA	mimG	A	CCGGG	UUGU	C	CGGGG	s2UUCm	m1I	m1A	GU	CCCCG	CGGCGGC	A
L UAA	GCGGGGG	UG	CCCG	AG+	CA	GGUCA A	AGGG	G	GCGGA	CU	UAA	G	A	UCCGC	UGGCGUAGGCCmU*G	C	GUGGG	UUCm	m1I	m1A	AU	CCCAC	CCCCCGC	A
L CAA	GCGGGGG	UG	CCCG	AG+	CU	GGUCA A	AGGG	G	GCGGA	CU	CAA	G	A	UCCGC	UGGCGAAGGCCUAm	m5C	GCGGG	UUC	A	A	AU	CCCGU	CCCCCGC	A
S UGA	GCCGGGG	UG	CCCG	AG	C	GGUCC A	AGGG	*G	m5CUGGC	CU	mchm5UmGA	mimG	A	GCCAG	UAGGGUUAUCCCU*G	C	GUmGGG	s2UUC	A	A	AU	CCCAC	CCCCGGC	G
S GGA	GCCGGGG	UG	CCCG	AG	U	GGACU A	AGGG	G	CUGGC	CU	GGA	G	A	GCCAG	UGUGGAUCUUCCACG	C	GCGGG	UUC	A	A	AU	CCCGC	CCCCGGC	G
Y GUA	CCCGCCG	UA	GCUC	AG+	CCC	GGU G	GAGC	*Gm	CCCGG	CU	GUA	mimG	A	CCGGG	UGGU	C	CGGmGG	s2UUCm	m1I	m1A	GU	CCCCG	CGGCGGG	A
W CCA	GGGCCCA	Um1A	m2GCUC	AG+	CCA	GGU A	GAGU	G	CCGGG	CmU	CmCA	mimG	A	CmC*CGG	UGGU	C	CGGGG	UUC	A	A	GU	CCCCG	UGGGCCC	A
Mi CAU	AGCGGCG	Um1A	m2GGGA	AG+	CCU	GGU A	UCCC	*G	CAGmGG	CmU	CAU	A	A	CCCUG	AGGU	C	CCUmGG	s2UUCm	m1I	m1A	AU	CCmAGG	CGCCGCU	A
N GUU	GCCGCGG	UA	GCUC	AG	UU	GGC A	GAGC	G	CCGGG	CU	GUU	A	A	CCCGG	UGGU	C	GGUGG	UCC	G	A	GU	CCACC	CCGCGGC	G
T GGU	GCCGCGG	UA	GCUC	AG	CCU	GGCU A	GAGC	*G	UUGCC	CU	GGU	t6A	A	GGCAA	AGGU	C	CCGGG	UUC	A	A	AU	CCCGG	CCGCGGC	U
	1	8	1	1			2	2	2	3				3	4	4	4	5				6	6	7
			0	4			2	6	7	2				9	4	8	9	4				1	6	3
	....	UR	G..Y	AG		GG R	...Y	G	...G.	CU				.C...		C	....G	UUC	R	m1A	RY	C....	....	R

Figure S10

Structural alignments of the purified and analyzed tRNAs from each species. The secondary structural elements of the tRNA cloverleaf are indicated above and below each alignment with key positions numbered following the standard nomenclature of yeast tRNA-Phe. On the last line, highly conserved residues are indicated. For clarity, the base-paired stems are colored (yellow for the AA-stem; green for the D-stem; cyan for the AC-stem; purple for the TΨC-stem throughout the alignments). For the long-arm tRNAs, tRNA-Ser and tRNA-Leu, the possible pairings of the additional helix are in italics. The highly conserved residues are in bold and those quasi-conserved not bolded in the consensus lines below each alignment. Square brackets are used when there is a potential ambiguity in nomenclature. The amino acids are arranged alphabetically with at the top (in blue) those coded by CG-rich codons (1st group: C1-quadrant corresponding to G36-containing tRNAs – Figure 2); 2nd group: G1-quadrant corresponding to C36-containing tRNAs) and below (in red) those coded by UA-rich codons (1st group: U1-quadrant corresponding to A36-containing tRNAs; 2nd group: A1-quadrant corresponding to U36-containing tRNAs - Figure 2). The code for modified nucleosides follows the one in MODOMICS (Boccaletto et al. 2018). Thus, the modification is always indicated in front of the letter with the atom number of the base modified (m5C, s2U, m1G or m5s2U etc.). The exception is 2'O-methylation on the ribose indicated after the base without atom number (Gm, Cm, …). Because, we cannot always assign the methylation position, we underline such residues (for example, Gm means the nucleotide could be either m1G, m2G... or Gm). Brackets are used when there is a mixture of modifications, e.g. [A/Am] means some of those As are methylated on the O2' and [A/Am] means that the methylation position of the A is unknown. For shortness, we introduced here: ac6A = *A; ac4C = *C; m22G = *G. An "x" before a nucleotide, e.g. xG, or after a modification symbol, e.g. m^x, means that the modification has not been formally identified (see text). Pseudouridines are not represented, except when the available evidence points formally to their presence, as for position 54 (the code MODOMICS is "Y").

Figure S11

Drawings of tertiary pairs discussed in the text. A: the *trans* Watson-Crick/Watson-Crick G15/C48 (from PDB 1EHZ). B: the *trans* Watson-Crick/Hoogsteen T54/m1A58 (from PDB 1EHZ). C: the C32/A38N contact (from PDB 1EHZ). D: the G26/A44 base pair (from PDB 1EHZ). Bottom right: the *trans* Watson-Crick/Hoogsteen A54/m1A58 (from PDB 1YFG) seen from the top (above) and along the m22G26 (below). Drawings made using Pymol (PyMOL(TM) 1.7.7.6 – Incentive Product Copyright © Schrodinger LLC.

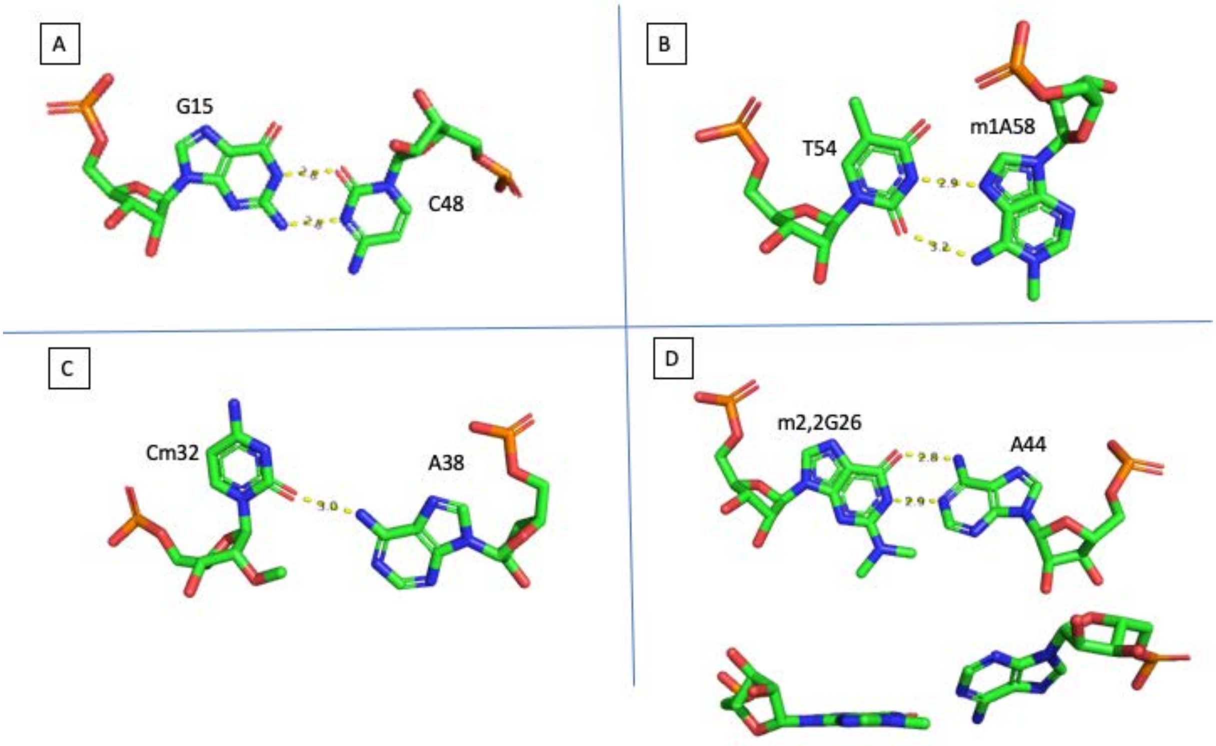

Figure S12

Contacts between the P-site tRNA and the large rRNA in bacterial ribosome (A and B) (from Watson et al. 2020). The contacts occur in the minor groove of base pairs 29-41 and 30-40 in the anticodon stem. Notice how the hydroxyl groups of 40 and 41 are forming H-bonds locking in those residues. Further A1338 forms a *trans* Hoogsteen/Watson-Crick pair (a "sheared" base pair) with G944. C: The environment around residues 59 and 60 (from PDB 2DU3). D: The H-bond between O2'(A37) and N1(A1913) (from Watson et al. 2020). Drawings made using Pymol (PyMOL(TM) 1.7.7.6 – Incentive Product Copyright © Schrodinger LLC.

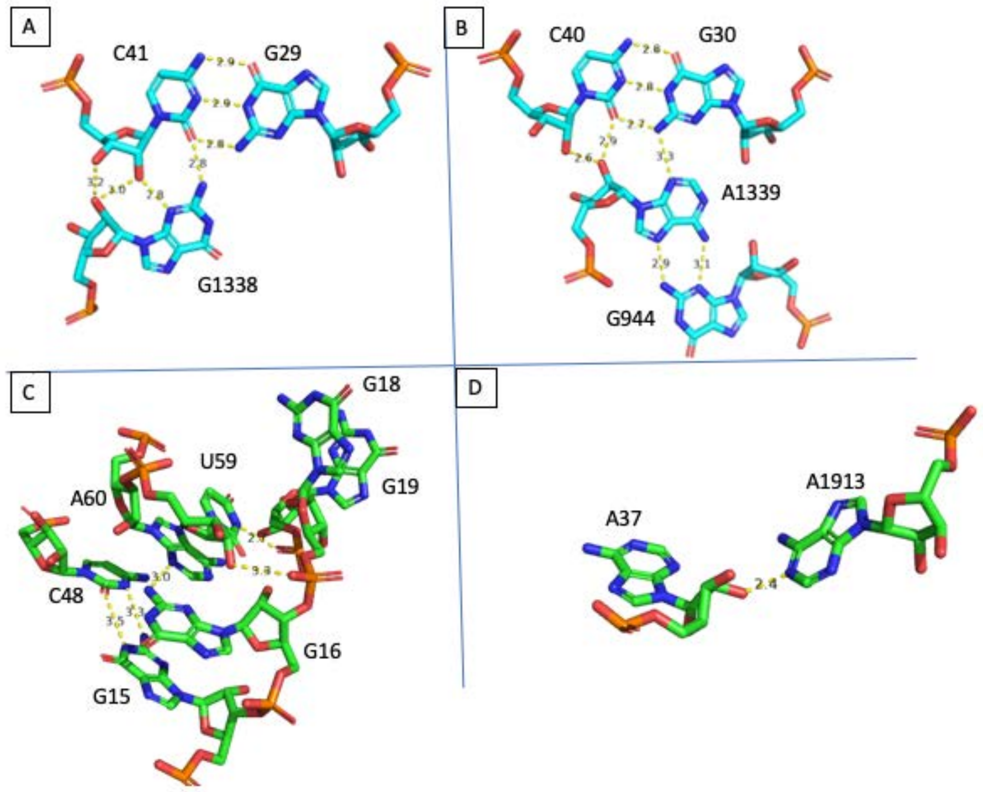

Table S1

Oligonucleotide fragments found in RNase digests that contain a methylation. In the case of RNase T1, m2G and m22G could be cleaved (depending tRNA as well as on experimental conditions) but m7G, m1G and Gm was never cleaved. In the case of RNase A, m5C and m1Y are cleaved and Cm, Um, m5U and m5s2U are not cleaved.

m/z	z	Molecular mass (Da)	Sequence	tRNA	Modification	T1 cleavage
M. maripaludis						
719,13	2	1439,3	A[Gm]AGp + NA + K	Ala UGC	[Gm] 22	no
1070,47	3	3213,4	CCCU[mnm5U]UC[m1G]AG>p	Glu UUC	[m1G] 37	no
1135,7	2	2272,4	GmAUCCUGp	Pro UGG	[m1G] 37	no
1440,64	2	2882,3	GmAUCCCUGp	His GUG	[m1G] 37	no
1154,21	2	2309,4	AAC[m2,2G]CUGp	Phe GAA	[m2,2G] 26	no
667,11	2	1335,2	AAC[m2,2G]>p	Tyr GUA	[m2,2G] 26	no
824,62	2	1650,2	C[m2,2G]CCGp	Ser UGA	[m2,2G] 26	no
827,64	2	1656,3	C[m2,2G]ACG>p	Asn GUU	[m2,2G] 26	no
968,62	2	1938,2	C[m2,2G]CCUG>p	Lys UUU	[m2,2G] 26	no
968,67	2	1938,3	C[m2,2G]CCUG>p	Thr GGU	[m2,2G] 26	no
977,61	2	1956,2	C[m2,2G]CCUGp	Lys UUU	[m2,2G] 26	no
978,17	2	1957,3	U[m2,2G]CUCGp	Ile	[m2,2G] 26	no
980,68	2	1962,4	C[m2,2G]UACG>p	Ser GGA	[m2,2G] 26	no
1001,62	2	2004,2	AU[m2,2G]CAGp	Leu UAG	[m2,2G] 26	no
P. furiosus						
856,14	2	1713,3	[m2,2G]AU[Am]Gp	Cys GCA	[m2,2G] 6	no
684,1	2	1369,2	C[ac4C][m2,2G]Gp	Ala UGC	[m2,2G] 6	no
679,13	2	1359,3	C[m2,2Gm]CGp	Leu CAG	[m2,2Gm] 26	no
822,61	2	1646,2	C[m2,2Gm]CCG>p	Ala UGC	[m2,2G] 26	no
824,64	2	1650,3	C[m2,2G]CCGp	Leu CAG	[m2,2G] 26	no
857,65	2	1716,3	AC[ac4C][m2,2G]Gp	Arg CCU	[m2,2G] 26	no
834,64	2	1670,3	C[m2,2Gm]CCGp	Ala UGC	[m2,2G] 26	no
729,13	2	1459,3	A[mimG]A[m2,2G]p	Ser GGA	[m2,2G] 39	**yes**
843,64	2	1688,3	A[m5C][m5C][Gm]Gp	Gln CUG	[Gm] 50	no
1039,15	1	1039,1	[m2,7Gm]UG>p	Glu CUC	[m2,7Gm] 10	no
1209,66	2	2420,3	p[m7G]CCC[ac4C][m2,2G]>p	Glu CUC	[m7G] 10	no
S. acidocaldarius						
1291,5	2	3876,5	UC[m2G]UCUAG+C[Cm]UG>p	Val GAC	[m2G] 10	no
1134,17	2	2269,3	CCCAU[Am][m2G]p	Trp CCA	[m2G] 10	**yes**
1100,48	2	3303,4	UAm[m22G]UCUAG+CG>p	Asp GUC	[m22G] 10	no
1328,69	2	2658,4	UCUAG+C[Gm]Gp	Gln UUG	[Gm] 18	no
1432,89	2	4300,7	UA[m22G]UAUAG+CCC[Gm]Gp	Glu UUC	[Gm] 19	no
1154,16	2	2309,3	GmAUCmCAGp	Gln UUG	[Gm] 23	no
670,6	2	1342,2	[m2,2G][Cm]UG>p	Ser UGA	[m2,2G] 26	no
672,6	2	1346,2	U[m2,2G]CGp	Ala GGC	[m2,2G] 26	no
816,61	2	1634,2	C[m2,2G]UUGp	Thr GGU	[m2,2G] 26	no
1142,13	2	2285,3	AC[m2,2G]CCUGp	Gly GCC	[m2,2G] 26	no
1154,17	2	2309,3	AU[m2,2G]CCAGp	Pro UGG	[m2,2G] 26	no
984,15	2	1969,3	Cm[m2,2G]CCCGp	Phe TRUE	[m2,2G] 26	no
987,64	2	1976,2	ACm[m2,2G]CUG>p	Val GAC	[m2,2G] 26	no
832,13	2	1665,3	U[m2,2Gm]CCGp	Trp CCA	[m2,2Gm] 26	no
984,13	2	1969,3	C[m2,2Gm]CCCGp	Tyr GUA	[m2,2Gm] 26	no
820,61	2	1642,2	AC[m1G]CG>p	Val GAC	[m1G] 37	no
1123,12	2	2247,2	[m1G]CCCCUGp	Gln UUG	[m1G] 37	no
961,63	2	1924,3	[m1G]CCCUG>p	Pro UGG	[m1G] 37	no
961,63	2	1924,3	[m1G]CUCCG>p	Leu GAG	[m1G] 37	no
975,64	2	1952,3	CCmU[m2,2G]CG>p	Leu UAA	[m2,2G] 47	no
1162,78	3	3490,3	UUAUCCCU[m2,2G]CG>p	Ser UGA	[m2,2G] 47	no

m/z	z	Molecular mass (Da)	sequence	tRNA	Modification	RNase A cleavage
M. maripaludis						
821,10	2	1643,2	GGA[Cm]U>p	Phe GAA	[Cm]32	no
1030,64	2	2062,3	GGGGG[m1Y]p	Arg UCU	[m1Y]54	**yes**
1030,61	2	2062,2	GGGGG[m1Y]p	Ser UGA	[m1Y]54	**yes**
819,63	2	1640,3	[Cm][m1I][m1A]AU>p	Ser UGA	[Cm]56	no
820,12	2	1641,2	[Cm]G[m1A]AU>p	Ile GAU	[Cm]56	no
P. furiosus						
691,13	1	691,1	[m2,2G][m5C]>p	Met CAU	[m5C] 27	**yes**
1030,16	2	2061,3	[Cm]AU[m6t6A]AC>p	Met CAU	[Cm]32	no
1014,16	2	2029,3	GGAGA[m5C]p	Gly GCC	[m5C]48	**yes**
846,60	2	1694,2	GGG[m5U]Up	Cys GCA	[m5U]54	no
1019,12	2	2039,2	GGGG[m5U]Up	Met CAU	[m5U]54	no
828,63	2	1658,3	[Cm][m1I][m1A]Up	Gly GCC	[Cm]56	no
1000,66	2	2002,3	[Cm][m1I][m1A]AGCp	Thr GGU	[Cm]56	no
1001,16	2	2003,3	[Cm]G[m1A]AGCp	Met CAU	[Cm]56	no
854,64	2	1710,3	A[mimG]A[Cm]Cp	Trp CCA	[Cm]39	no
1016,18	2	2033,3	[Cm][Gm]GGAC[m5C]p	Trp CCA	[Cm]41	no
1016,18	2	2033,3	[Cm][Gm]GGAC[m5C]p	Trp CCA	[m5C]48	**yes**
1014,13	2	2029,3	GGAGA[m5C]p	Gly GCC	[m5C]48	**yes**
897,46	2	2694,4	AGGAG[Um][Gm]Cp	Ini	[Um] 21	no
1011,12	2	2023,2	GAGG[m5s2U]Up	Ini	[m5s2U]54	no
S. acidocaldarius						
819,62	2	1640,2	[Cm]O[Am]AU>p	Met CAU	[Cm]56	no
827,09	2	1655,2	[Um]GG[s2U]Up	Met CAU	[Um] 51	no
828,62	2	1658,2	[Cm]O[Am]AUp	Gly GCC	[Cm]56	no
830,12	2	1661,2	GGA[Cm]Up	Leu GAG	[Cm]32	no
836,62	2	1674,2	[Cm][m1I][Am]GUp	Tyr GUA	[Cm]32	no
836,63	2	1674,3	[Cm]AG[m1G]Cp	Leu CAG	[Cm]34	no
844,62	2	1690,2	G[Cm]GG[m5C]p	Asp GUC	[Cm]69	no
844,62	2	1690,2	G[Cm]GG[m5C]p	Asp GUC	[m5C]72	**yes**
854,64	2	1710,3	A[mimG]A[Cm]Cp	Trp CCA	[Cm]39	no
999,11	2	1999,2	[Cm]GGGs2UUp	Gly GCC	[Cm] 50	no
999,62	2	2000,2	[Um]GGGs2UUp	Val GAC	[Um] 50	no
1006,13	2	2013,3	[Cm]GmGGs2UUp	Asp GUC	[Cm]50	no
1009,65	2	2020,3	A[Gm]GG[Cm]Up	Met	[Cm]32	no
1066,12	2	2133,2	GG[Cm]G[xG]Up	Gly GCC	[Cm]50	no
1131,83	3	3397,5	GGGAGAG[Cm][m2,2G]Cp	Phe GAA	[Cm]25	no
1172,13	2	2345,3	G[Um]GGGs2UUp	Leu GAG	[Um] 50	no
1172,13	2	2345,3	G[Um]GGG[s2U]Up	Leu CAG	[Um] 50	no
1172,14	2	2345,3	G[Um]GGGs2UUp	Ser UGA	[Um] 50	no
1200,68	2	2402,4	AAGGG[m2,2G][m5C]p	Ser UGA	[m5C]27	**yes**
1247,18	3	3743,5	AGGA[Um][m2,2G]GGGGCp	Asp GUC	[Um] 25	no

Table S2

Types and positions of chemical modifications of nucleotides in naturally occurring tRNAs from selected Archaea and their corresponding modification enzymes.

The compilation includes data from tRNAs purified from: two Methanococcales, i.e. the mesophile *Methanococcus maripaludis* (this work), and the hyperthermophile *Methanocaldococcus jannaschii* (Yu et al. 2019); three closely related hyperthermophiles from the Pyrococcale genus, i.e. *Pyrococcus furiosus* (this work), *Pyrococcus abyssi* and *Pyrococcus horikoshii* together with data of the unique tRNA-Trp from *Thermococcus kodakarensis* analyzed so far. They all belong to the Euryarchaeal subgroup of Archaea. The last group of tRNAs analyzed belongs to the Sulfolobales phylum of the Crenoarchaeota subgroup: *Sulfolobus acidocaldarius* (this work), *Sulfolobus solfataricus* and *Sulfolobus tokadai*. When known, are also listed the conventional acronyms of corresponding enzymes, for which the specificity has been experimentally validated, the group of ortholog genes they belong to (COG) and eventually the precise genomic orf (when known) obtained from MODOMICS (https://iimcb.genesilico.pl/modomics/) or PubMed/Medline. The associated reference list corresponds to the selection of only one, often among many other valuable ones, that best describes the tRNA(s) or the modification enzyme(s). Some modifications are catalyzed by stand-alone enzymes while others, especially in hyperthermophilic archaea, result from more complexed enzymatic machinery guided by sRNA (aFlpA C/D box sRNP in the case of 2'-O-methylations and Cbf5 H/ACA sRNP in the case of isomerisation of U into pseudouridine). In bold are the new modifications, observed in the present study, and for which the corresponding enzymes are not yet known. When underlined [<u>X</u>- methylated], it means that the nucleotide X is monomethylated but we do not know yet whether it is on the base or on the 2'O-ribose. xU, and xG correspond to yet undetermined chemical modifications of the base that do not appear to correspond to any modification identified so far. Together with all the modified nucleotides reported in this work, are also listed those not indicated in Table 1 and Figure 3 but that have been experimentally identified in independent works, the archaeal species, the gene coding for the corresponding enzyme with references are clearly mentioned. Numbering positions of nucleotides in tRNA are the conventional ones used in the tRNA data bank (http://trnadb.bioinf.uni-leipzig.de).

	MODOMICS of archaeal tRNA		Methanococcales			
Pos	MODS	ENZYME (COG)	Orf	Archaea genus	References	Pmid
6	m2G	TrmN/Trm14 (COG0116)	MJ0438	*M. jannaschii*	Menezes et al, 2011	.21693558
**7**	**ac6A**(see text)	**unknown**		***M. maripaludis***	**this work**	
8	s4U	ThiI (COG0301)	Mmp1354/Mj0931	*M. maripal/jann*	Liu et al, 2012	.22904325
10	m2G/m2,2G	Trm-G10 (COG1041)	Mmp0149/Mj0710	*M. maripal/jann*	Yu et al, 2019	.30745370
15	G+	TgtA + (arcS) (COG1549)	Mj0436 +(Mj1022)	*M. Jannaschii*	Bai et al, 2000	.10862614
15	G+	TgtA + (arcS) (COG1549)	Mmp0610+(Mj1595	*M. maripaludis*	Phillips et al, 2012	.22032275
**17**	**s2C**	**unknown**		***M. maripaludis***	**this work**	
**22**	**Gm**	**unknown**		***M. marIpaludis***	**this work**	
26	m2G/m2,2G	Trm1 (COG1867)	Mmp0228/Mj0946	*M. maripal/jann*	Graham/Kramer, 2007	.17673084
32	s2C	*TtcA-like* (COG0037)	*Mmp1356/Mj1157*	*M. maripal/jann*	Yu et al, 2019	.30745370
32	Cm/Um (+34?)	*FlpA +snoRNP* (COG1889)	Mmp0597/Mj0697	*M. maripal/jann*	Yu et al, 2019	.30745370
**33**	**s2U**	**unknown**		***M. maripaludis***	**this work**	
34	C+	TiaS (COG1571)	Mmp0616/Mj1095	*M. maripal/jann*	Yu et al, 2019	.30745370
34	mcm5(s2)U	*Elp3-like* (COG1243)	*Mmp1577/Mj1136*	*M. maripal/jann*	Huang et al, 2008	.18755837
34	s2(mnm5)U	Ncs6/Ctu1-like+(**unknown**)	Mmp1356/Mj1157	*M. maripal/jann*	Liu et al, 2014	.24530533
34	se2(mnm5)U	*ybbB-like*/selU+(**unknown**)	*Mmp0899/Mj0053*	*M. maripal/jann*	Su et al, 2012	.22293502
34	s2(cnm5)U	Ncs6 + (**unknown**)		*M. maripal/jann*	Yu et al, 2019	.30745370
34	Cm (+32?)	*FlpA +snoRNP* (COG1889)	*Mmp0597/Mj0697*	*M. maripal/jann*	Singh et al, 2004	.15347671
**34**	**xU(cnU)** see text	**unknown**		***M. maripaludis***	**this work**	
37	t6A	Tsa2+TsaB/D/E	Mj0062 + Mj1130	*M. Jannashii*	Wan et al, 2016	.27302132
37		(t6A complex)	Mj0594 + Mj0186	*M. Jannaschii*		
37	t6A	Tsa2+TsaB/D/E	Mmp0186 + 0415	*M. maripaludis*	Thiaville et al, 2014	.25629598
37		(t6A complex)	Mmp0247 + 0897	*M. maripaludis*		
37	ms2t6A	MtaB (+ t6A complex)	Mmp0412/Mj0867	*M. maripal/jann*	Yu et al, 2019	.30745370
37	ms2hn6A	(MtaB) + hn6A complex	*t6A-related enzymo*	*M. maripal/jann*	Reddy et al, 1992	.1280806
37	m1G	Trm5b (COG2520)	Mmp0323/Mj0883	*M. maripal/jann*	Go-Ito et al, 2008	.18384044
37	imG-14	(Trm5b)+Taw1 (COC0731)	Mmp1440/Mj0257	*M. maripal/jann*	Yu et al, 2019	.30745370
37	imG	(Trm5b+Taw1)+Taw3	Mmp0310/Mj1510	*M. maripal/jann*	Yu et al, 2019	.30745370
37	yW	(Trm5+Taw1)+Trm2(+Taw3)	Mmp0560/Mj1557	*M. maripal/jann*	Umitsu et al, 2009	.19717466
37	mimG	Trm5b+Taw1+Trm5a+Taw3		*M. maripal/jann*	deCrecy-Lagard et al,2010	.20382657
**37**	**xG** (see text)	*wyosine metabolism*		***M. maripaludis***	**this work**	
**37**	**f6A** (see text)	**unknown** *(or TrmM-like)*		***M. maripaludis***	(Golovina et al, 2016)	.26707202
**39**	**s2U**	**unknown**		***M. maripaludis***	**this work**	
48	m5C (+49)	Trm4 (COG0144)	Mmp1126/Mj0026	*M. maripal/jann*	Kuratani et al, 2010	.20600111
54	Psi (+55)	Pus10 (COG1258)	Mmp0924/Mj0041	*M. maripal/jann*	Gurha & Gupta, 2008	.18952823
54	m1Psi	(Pus10) >>TrmY(COG1901)	Mmp0094/Mj1640	*M. maripal/jann*	Chatterjee et al, 2012	.22274953
54	s4(m1Psi)	TtuA + (TtuB) (COG0037)	Mj1478	*M. jannaschii*	Shigi, 2014	.24765101
55	Psi	*probably present* (Pus10)		*M. maripal/jann*	Fitzek et al, 2018	.29349599
56	Cm	Trm56 (COG1303)	Mmp1425/Mj1385	*M. maripal/jann*	Yu et al, 2019	.30745370
57	(m1A) >> m1I	(TrmI) >> + **unknown**		*H. volcanii*	Grosjean et al, 1996	.7501451
58	m1A	TrmI (COG2519)	Mmp1375/Mj0134	*M. maripal/jann*	Graham & Kramer, 2007	.17673084
67	m2G	**unknown**		*M. jannaschii*	Yu et al, 2019	.30745370

Pos	MODS	ENZYME (COG)	Orf	Archaea genus	References	Pmid
	**MODOMICS of archaeal tRNA**			**Pyrococcales**		
**1**	**2H-m7G** (see text	**unknown**		***P. furiosus***	**this work**	
**5**	**ac4C**	**unknown**		***P. furiosus***	**this work**	
6	m2G/m22G	TrmN/Trm14 (COG0116)	PF1002	***P. furiosus***	Fislage et al, 2012	.22362751
	Cm-6 / **Um-8**	**unknown/unknown**		*T. kodak/**furios***	Hirata et al, 2019	.31405913
8	s4U-8(+9)	ThiI (COG0301)	PF1288/TK0368	*P. furios/kodak*	Cavuzic & Liu, 2017	.28287455
9	m1A/m1G	Trm10 (COG2419)	PF0678/TK1257	*P . furios/kodak*	Krihnamohan et al, 2019	.30704107
10	m2G/m2,2G	Trm-G10 (COG1041)	PF0400/TK0980	*P. furios/kodak*	Armengaud et al, 2004	.15210688
**10**	**2H-m27Gm**(text)	**unknown**		***P. furiosus***	**this work**	
15	G+	TgtA + (arcS) (COG1549)	PF0071/TK2156	*P. furios/horik*	Ishitani et al, 2002	.12054814
17	s2C	**unknown**		*T. furios/kodak*	Hirata et al, 2019	.31405913
**18**	**Gm**	*FlpA +sRNP (or TrmH-like?)*	*PF0059*	***P. furiosus***	**this work**	
	**Um-21**; **Gm**-22	**unknown** *or FlpA +sRNP*		*T. kodak/**furios***	Hirata et al, 2019	.31405913
26	m2G/m2,2G(m)	Trm1(+**unknown**)(COG1867	PF1871/TK0970	*P. furios/kodaka*	Constantinesco et al,1998	.9685492
32	s2C	*TtcA-like* (COG0037)	*PF0273/TK1821*	*P. furios/kodaka*	Cavuzic & Liu, 2017	.28287455
32	m5C(m)	**unknown** *(+FlpA-snoRNA)*		*T. kodakarensis*	Hirata et al, 2019	.31405913
34	C+	TiaS (COG1571)	PF1855/TK0553	*P. furios/kodak*	Ikeuchi et al, 2010	.20139989
34	s2(mnm5)U	Ncs6/Ctu1-like (+**unknown**)	PF0273/TK1821	*P. furios/kodak*	Cavuzic and Liu, 2018	.28287455
34	(s2)mnm5U	Elp3-like (COG1243)				
34	Cm/Um(+39)	FlpA + Nop5 + snoRNA	PF0059/TK0183	*P. furios/kodak*	Clouet-d'Orval et al, 2001	.11713301
34	cnm5U	**unknown**		*Euryarchaeota*	see Mandal et al, 2014	.24344322
**34**	ac4C(m)	*TmcA-like(+FlpA)* (COG1444)	PF0504/TK0754	*P. furios/kodak*	(Bortolin et al, 2003)	.14602911
37	t6A	Tsa2 (+TsaB/D/E)	PF0306/TK0948	*P. furios/kodak*	Perrochia et al, 2012	.23258706
37	ms2(hn6A)	*MtaB-like* (+**unknown**)	*PF1912/TK2064*	*P. furios/kodak*	Reddy et al, 1992	.1280806
37	m1G	Trm5b (COG2520)	PF1415/TK0497	*P. furios/kodak*	Wu et al, 2017	.28911863
37	imG2	(Trm5a+Taw1) + Taw22	PF0089/TK2223	*P. furios/kodak*	Urbonavicius et al, 2014	.24837075
37	mimG	(Trm5b+Taw1+ 22) +Taw3	PF2034/TK0175	*P. furios/kodak*	Perche-Letuvée et al,2012	.23043105
37	yW-86	(Trm5b+Taw1) + Taw2	PF0500/TK0071	*P. furios/kodak*	Umitsu et al, 2009	.19717466
**38**	**Am**	*probably FlpA + snoRNP*		***P. furiosus***	Singh al 2004	.15347671
**39**	**m22G**	**unknown**		***P. furiosus***	**this work**	
39	Cm (+34)	FlpA-Nop5+snoRNP	PF0059/TK0183	*P. furios/kodak*	Bortolin et al, 2003	.14602911
	Cm-41/42,Gm-42	*probably all FlpA + snoRNP*		*T. kodak/**furios***	Hirata et al, 2019	.31405913
**42**	**ac4C**	**unknown**		***P. furiosus***	**this work**	
**44**	**Um**	**unknown**		***P. furiosus***	**this work**	
**47**	**xU** (see text)	**unknown**		***P. furiosus***	**this work**	
48	m5C +49 (+27)	Trm4/**unknow-27**(COG0144	PF1553/TK0360	*P.furios/kodak*	Auxilien et al, 2017	.17470432
**50**	**Gm**	**unknown**		***P. furiosus***	**this work**	
54	s2(m5U)	TtuA (+TrmU54) (COG2265)	PF1172/TK1116	*P. furios/kodak*	Rose et al, 2020	.32459340
55	Psi (+54)	Pus10 (COG1258)	PF1139/TK0903	*P. furios/kodak*	Gurha & Gupta, 2008	.18952823
55	Psi	Cbf5 (+Gar1; no gRNA)	PF1785/TK1509	*P. furios/kodak*	Müller et al, 2007	.17704128
56	Cm	Trm56 *(or FlpA + snoRNP)*	PF0461/TK0060	*P.furios/kodak*	Renalier et al, 2005	.15987815
57	m1A (+58)	TrmI (COG2519)	PF1896/TK1328	*P. furios/kodak*	Roovers et al, 2004	.14739239
57	(m1A) >> m1I	(TrmI) >> + **unknown**		***P. furiosus***	Grosjean et al, 1999	.9973619
**63**	ac4C (+64)	**unknown**		***P. furiosus***	**this work**	
	m2G-67, **Gm**-68	**unknown-67/68**		*T. kodak/**P.furios***	Hirata et al, 2019	.31405913
72	m5C	NSun6 (COG0144)	PF0169/TK2122	*P. furios/kodak*	Li et al, 2019	.30541086

	**MODOMICS of archaeal tRNA**			**Sulfolobales**		
**Pos**	**MODS**	**ENZYME (COG)**	**Orf**	**Archaea genus**	**References**	**Pmid**
**3**	**ac4C**	**unknown**		***S. acidocaldarius***	**this work**	
9	m1A	Trm10 (COG2419)	Saci_1677	*S. acidocaldarius*	Kempenaers et al, 2010	.20525789
10	m2G/m2,2G	Trm-G10 (COG1041)	Saci_1283	*S. acido/solfata*	Wagner et al, 2004	.17150579
15	G+	TgtA+*ArcS-like* (COG0343)	Saci0659+*Saci0657*	*S. acido/solfata*	Phillips et al, 2012	.22032275
**17**	**<u>C/A</u>** methylated	**unknown**		***S. acidocaldarius***	**this work**	
18	Gm (+34)	FlpA+ Nop5 + snoRNP	Saci_1346	*S. acido/solfata*	Omer et al, 2006	.16861619
**20**	**<u>A</u>** methylated	**unknown**		***S. acidocaldarius***	**this work**	
**23**	**Gm**	*probably FlpA+Nop5+snoRN*	*Saci_1346*	***S. acidocaldarius***	**this work**	
**25**	**Cm/Um**	*probably FlpA+Nop5+snoRN*	*Saci_1346*	***S. acidocaldarius***	**this work**	
26	m2G/m2,2G(m)	Trm1(+unknown)(COG1867)	Saci_1343	*S. acidocaldarius*	≈ ubiquitous in Archaea	
**27**	**m5C**	**unknown**		***S. acidocaldarius***	**this work**	
**29**	**Gm**	**unknown**		***S. acidocaldarius***	**this work**	
32	Cm	TrmJ (COG0565)	Saci_0621	*S. acidocaldarius*	Somme et al, 2014	.24951554
34	C+	TiaS (COG1571)	Saci_0732	*S. acido/solfata*	Mandal et al, 2010	.20133752
34	Um/Cm (+18)	FlpA-Nop5 + snoRNP	Saci_1346	*S. acido/solfata*	Ziesche et al, 2004	.15522081
34	s2U(m)	*Ncs6/Ctu1-like* (COG0037)	*Saci_1570*	*S. acidocaldarius*	Liu et al, 2016	.27791189
**34**	**mchm5Um**	*Elp3-like* (COG1243)	*Saci_1044*	***S. acidocaldarius***	**this work**	
37	t6A	TsaC+ (Tsc complex)	Saci_1642+Saci0851	*S. tokadai*	Kuratani et al, 2011	.21538543
37	m1G	Trm5c (COG2520)	Saci_0559	*S. acidocaldarius*	Urbonavicius et al, 2016	.27852927
37	imG-14	(Trm5c) +Taw1 (COG0731)	Scai_1331	*S. acidocaldarius*	Urbonavicius et al, 2016	.27852927
37	imG2	(Trm5c +Taw1) +Trm5a	Scai_0030	*S. acido/solfata*	deCrecy-Lagard et al,2010	.20382657
37	mimG	(Trm5c +Taw2) +Taw3	Saci_1603	*S. acidocaldarius*	McCloskey et al, 1987	.27932585
39	Cm	*probably FlpA+Nop5+snoRN*	*Saci_1346*	*S. acidocaldarius*	see in *P. furiosus*	
40	m5C	*probably Trm4 (like in HVO)*		*S. acidocaldarius*	Grosjean et al, 2008	.18844986
**41**	**ac4C** (+40)	**unknown**		***S. acidocaldarius***	**this work**	
**47**	**<u>A</u>** methylated	**unknown**		***S. acidocaldarius***	**this work**	
**47**	**m2,2G**	**unknown**		***S. acidocaldarius***	**this work**	
**47**	**xU** (see text)	**unknown**		***S. acidocaldarius***	**this work**	
48	m5C (+49)	Trm4 (COG0144)	Saci_0717	*S. acidocaldarius*	almost in all Archaea	
50	Um/Cm (+18)	FlpA-Nop5+snoRNP	Saci_1346	*S. acidocaldarius*	Tang et al, 2005	.15659164
51	Gm	FlpA-Nop5+snoRNP		*S. acidocaldarius*	Zago et al, 2005	.15752202
54	Um?? > see text	***unknown***		*S. acidocaldarius*	Kuchino et al, 1982	.6178978
54	**s2U/s4Psi?**	TtuA+TtuB *see text*	Saci_0378+Saic1570	*S. acidocaldarius*	Rose et al, 2020	.32459340
54	**<u>mxsxU/Psi</u>** >text	**unknown**		*S. acidocaldarius*	**this work**	
55	Psi	*possibly Cbf5*	Scai_0811	*S. acidocaldarius*	Majumber et a, 2016	.27539785
56	Cm	Trm56 (COG1303)	Saci_1040	*S. acdocaldarius*	Renalier et al, 2005	.15987815
57	(m1A) >> m1I	(TrmI) + **unknown**		*S. acidocaldarius*	Yamaizumi et al, 1982	.7183961
58	m1A	TrmI (COG2519)	Saci_0844	*S. acidocaldarius*	Guelorget et al, 2011	.22168821
**62**	**<u>C</u>** methylated	**unknown**		***S. acidocaldarius***	**this work**	
**64**	**<u>U</u>** methylated	**unknown**		***S. acidocaldarius***	**this work**	
**69**	**<u>C</u>** methylated	**unknown**		***S. acidocaldarius***	**this work**	
72	m5C	NSun6 (COG0144)	Saci_1487	*S. acido/solfata*	Wagner et al, 2004	.17150579
**72**	**<u>U</u>** methylated	**unknown**		***S. acidocaldarius***	**this work**	
